# Addressing Research Needs in the Field of Plant Virus Ecology by Defining Knowledge Gaps and Developing Wild Dicot Study Systems

**DOI:** 10.3389/fmicb.2018.03305

**Published:** 2019-01-09

**Authors:** Tessa M. Shates, Penglin Sun, Carolyn M. Malmstrom, Chrysalyn Dominguez, Kerry E. Mauck

**Affiliations:** ^1^Department of Entomology, University of California, Riverside, Riverside, CA, United States; ^2^Department of Plant Biology, Michigan State University, East Lansing, MI, United States; ^3^Graduate Program in Ecology, Evolutionary Biology and Behavior, Michigan State University, East Lansing, MI, United States

**Keywords:** plant virus, crop-associated virus, dsRNA, metagenomics, perennial, agro-ecological interface, *Cucurbita*, *Datura*

## Abstract

Viruses are ubiquitous within all habitats that support cellular life and represent the most important emerging infectious diseases of plants. Despite this, it is only recently that we have begun to describe the ecological roles of plant viruses in unmanaged systems and the influence of ecosystem properties on virus evolution. We now know that wild plants frequently harbor infections by diverse virus species, but much remains to be learned about how viruses influence host traits and how hosts influence virus evolution and vector interactions. To identify knowledge gaps and suggest avenues for alleviating research deficits, we performed a quantitative synthesis of a representative sample of virus ecology literature, developed criteria for expanding the suite of pathosystems serving as models, and applied these criteria through a case study. We found significant gaps in the types of ecological systems studied, which merit more attention. In particular, there is a strong need for a greater diversity of logistically tractable, wild dicot perennial study systems suitable for experimental manipulations of infection status. Based on criteria developed from our quantitative synthesis, we evaluated three California native dicot perennials typically found in Mediterranean-climate plant communities as candidate models: *Cucurbita foetidissima* (buffalo gourd), *Cucurbita palmata* (coyote gourd), and *Datura wrightii* (sacred thorn-apple). We used Illumina sequencing and network analyses to characterize viromes and viral links among species, using samples taken from multiple individuals at two different reserves. We also compared our Illumina workflow with targeted RT-PCR detection assays of varying costs. To make this process accessible to ecologists looking to incorporate virology into existing studies, we describe our approach in detail and discuss advantages and challenges of different protocols. We also provide a bioinformatics workflow based on open-access tools with graphical user interfaces. Our study provides evidence that dicot perennials in xeric habitats support multiple, asymptomatic infections by viruses known to be pathogenic in related crop hosts. Quantifying the impacts of these interactions on plant performance and virus epidemiology in our logistically tractable host systems will provide fundamental information about plant virus ecology outside of crop environments.

## Introduction

The integration of plant virology and ecology is recent, stimulated by the discovery that viruses are important but largely undescribed and understudied components of the ecology of wild plants. Since the inception of this field, ecologists and plant virologists have worked to illuminate two issues: (i) the ecological roles of plant viruses and vectors in unmanaged ecosystems, and (ii) the influence of ecosystem properties on the distribution and evolution of plant viruses and their vectors (Malmstrom et al., 2011). Thanks to these efforts, we now know that wild plants frequently harbor infections by a wide diversity of plant viruses, which almost certainly influence host traits in ways not previously quantified or considered by the field of plant ecology (Malmstrom and Alexander, 2016). Evidence further suggests that the characteristics of plant viruses can be influenced by selection in natural communities and across ag–wild and urban–wild interfaces (Rodelo-Urrego et al., 2015; Bernardo et al., 2018). But much remains to be learned about how ecosystem properties influence virus evolution and vector interactions.

The need for research in this area has never been greater. Among emerging plant diseases, viruses are the most common causal agents (Anderson et al., 2004). Natural areas have undergone significant fragmentation as agricultural production and urbanization has increased (Fischer and Lindenmayer, 2007; Ellis et al., 2010). A key issue is that the expanded array of agro-ecological boundaries has created new opportunities for microbes associated with wild and cultivated plants to interact and to move between host types. Thus, it is critical to ask to what degree these interactions will influence microbial evolution and the probability that some microbes will emerge as novel pathogens (Alexander et al., 2013; Roossinck and García-Arenal, 2015).

At present, systems-level understanding of plant virus dynamics across agro-ecological interfaces is hampered by a dearth of information from wild communities, including those that border agricultural and urban land cover. Historically, most monitoring of plant virus dynamics has focused on cropping systems (Wren et al., 2006; Alexander et al., 2013; Roossinck and García-Arenal, 2015), although new geo-metagenomics approaches have begun to reveal virus distributions within non-cultivated systems (Bernardo et al., 2018). Here, we demonstrate that additional knowledge constraints arise because understanding of plant–virus dynamics is skewed toward annual host systems, which are more dominant in agriculture than in nature. While viruses have been tracked in some perennial crops such as stone fruit and grapes (e.g., plum pox virus), similar studies are rare for long-lived perennials in nature, even though perennials are dominant in most natural communities and may harbor diverse infections (Alexander et al., 2017).

To advance understanding in these critical research areas, it is essential that virus interactions within non-cultivated perennial systems be investigated. Perennials have potential to serve as modulators of viral virulence by exerting selection for longer-term host survival, or, in other cases, as sites for virus recombination through accumulation of co-infections or even as reservoirs for novel disease agents. In turn, non-cultivated perennial systems may be perturbed and affected by microbial movement from agricultural crops, which may also serve as reservoirs and exert selection pressures on viruses in ways that are not fully recognized. From an ecological perspective, viruses from crop environments may modify the expression of host plant functional traits in ways that alter stress tolerance, fitness (Alexander et al., 2017), and competitive interactions (Malmstrom et al., 2017), with significant implications for conservation of threatened native plant communities.

To advance work in this area, several hurdles need to be surmounted. One need is to simplify methods of virus detection and identify likely pitfalls so that researchers without backgrounds in molecular virology can properly examine the viromes of wild plant study systems. A second need is to identify promising wild plant–virus systems that can serve as useful models for short- and long-term manipulative work that goes beyond surveys and correlational studies. For example, we found that a large fraction of plant virus ecology work involving factor manipulation has been conducted on Poaceae hosts in a single ecosystem type: invaded California grasslands (see “Results” section). There is thus pressing need to identify new model systems involving dicot plant hosts beyond *Arabidopsis*. In the study presented here, we conducted a review of plant virus ecology literature to quantify resource gaps in the field, then use these results to define criteria for selecting the most useful new systems for future work. We then present a case study of three potential model hosts to demonstrate how investigators with limited budgets might initially evaluate the viromes of candidate wild plant systems, and tractability of various detection assays, with the aim of incorporating virus dynamics into ecological studies.

**FIGURE 1:**
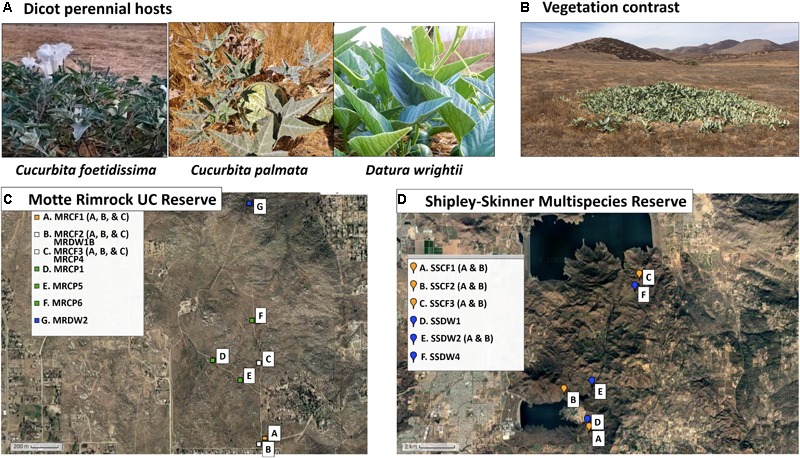
Study system and sampling locations. **(A)** Images of asymptomatic host plants sampled from the two reserve sites. **(B)** Typical growth habit of *C. foetidissima* during arid summer months. Green foliage contrasts strongly with soil and dry, senescent vegetation. **(C)** Satellite image map of populations sampled from the University of California Motte Rimrock Reserve. **(D)** Satellite image map of populations sampled from Shipley-Skinner Multispecies Reserve. Maps created by GPS Visualizer online.

The case study presented here focuses on fragmented semi-arid plant communities in southern California (United States), which is a Mediterranean-climate biodiversity hotspot (Myers et al., 2000). To explore the virus dynamics among keystone species within these communities, we used both Illumina sequencing and custom detection assays to study the viromes of three native dicot perennials [*Cucurbita foetidissima* and *Cucurbita palmata* (Cucurbitaceae), and *Datura wrightii* (Solanaceae)] that are summer-growing species within fragmented preserves of mixed grassland, xeric sage scrub and chaparral communities in the Southwestern United States (Figure 1A). Grassland, chaparral, and sage scrub habitats are the core native plant communities in Southern California and are dominated by perennials. Communities with these features, such as the Tallgrass Prairie Preserve in Oklahoma, have contributed significantly to our emerging understanding of virus biodiversity in wild plants (Muthukumar et al., 2009; Scheets et al., 2011; Min et al., 2012; Thapa et al., 2012, 2015). Efforts are underway to conserve and maintain these habitats, which persist as fragmented preserves between agricultural and urban environments.

For our study, we targeted two agriculture and urban-adjacent preserves in Riverside County, CA, United States, that feature these Mediterranean climate communities (Figure 1). Mediterranean-climate systems are characterized by cool winter growing seasons with some rain, followed by summer drought. Consistent with these seasonal patterns, the grassland, sage scrub, and chaparral environments within our selected sites include both winter- and summer-growing perennial species. Winter-growing species, while still adapted for xeric conditions, do not thrive in extreme heat and typically senesce by March or April each year. Summer species are drought- and heat-adapted plants capable of resisting severe abiotic stress. Due to these traits, the summer-growing species targeted in our study (Figure 1) are some of the few plants in leaf and bloom at the same time that insect vector populations peak in agricultural crops (Chu et al., 2001) and are likely exposed to both crop-associated and wild insect-transmitted viruses. Our summer-growing perennials also have congeneric and confamilial crop hosts grown in adjacent agricultural areas (squash and melons [Cucurbitaceae] and potatoes and peppers [Solanaceae]) and therefore may be susceptible to vectors and viral pathogens originating from monocultures of these crops. Thus, we hypothesized that we would frequently find infections by crop-associated viruses in our target species. We also hypothesized that these hosts could be important sources of novel emerging pathogens as infecting viruses evolve and adapt to exploit host tissues over multiple seasons. Therefore, we expected that if crop-associated viruses were present, some of them might have diverged substantially from those circulating in crop habitats while engaging in a persistent association with the same host individual across seasons. Finally, we expected to find novel plant viruses and viruses infecting plant fungal associates (endophytes, fungal pathogens), as these have been discovered in nearly all virus biodiversity studies to date that include steps for enrichment of virus nucleic acids followed by next-generation sequencing (NGS) (Roossinck, 2014; Stobbe and Roossinck, 2014).

## Materials and Methods

### Identifying Knowledge Gaps and Resource Needs in the Field of Virus Ecology

Rapid progress in the field of plant virus ecology was ignited by the founding of an international research consortium known as the Plant Virus Ecology Network (PVEN), which was established in 2007 by Ulrich Melcher and Carolyn Malmstrom with funding from the United States National Science Foundation (NSF). PVEN supported international workshops that were instrumental in connecting molecular virologists, epidemiologists, ecologists, entomologists, and other researchers with diverse skill sets relevant to plant virus ecology. NSF records indicate that PVEN stimulated the production of over 75 peer-reviewed publications between 2007 and 2011. To analyze the impact of PVEN on the field of virus ecology, and identify knowledge and resource gaps, we quantified the content of papers arising directly from PVEN support (**precursor papers**) and subsequent papers that cited these precursor papers (**product papers**). Precursor papers consisted of all peer-reviewed publications listed in the NSF reporting website (NSF Award Abstract #0639139, 2011), excluding conference abstracts. We categorized each precursor paper as contributing to 1–3 possible research areas from among the following: *Virus evolution, Virus discovery, Virus description, Virus effects on host traits, Virus–vector interactions, Epidemiology, Methods, Host resistance, Review papers, Environmental virology, and Theoretical* (mathematical modeling). A full description of the categories, as well as a complete list and categorization of the precursor papers, can be found in Supplementary Data Sheet 1. We designated six of these categories (*Virus evolution, Virus discovery, Virus effects on host traits, Virus–vector interactions, Epidemiology, and Environmental virology*) as core research categories of virus ecology based on previously defined criteria (Malmstrom et al., 2011).

For precursor papers within each of these six categories, we assessed the ecological focus of research content by categorizing the domestication status of plant hosts studied (crop, wild, or crop + wild), the plant life history strategy (annual, perennial, or both annuals and perennials), and the characteristics of the viruses studied [crop-associated (pathogenic viruses prevalent in crop systems), wild (only known to associate with non-crop hosts), or both crop-associated and wild]. To evaluate impact, we used Google Scholar to obtain a list of all subsequent papers (here, ‘product papers’) citing each precursor paper since its publication. Each product paper was evaluated to determine if the content matched one or more of the six core virus ecology research areas. The ecological focus of product papers was determined in the same manner as for precursor papers. We assessed both literature sets for biases in favor of certain host and virus characteristics to identify knowledge gaps and resource needs within the field of virus ecology. We also evaluated characteristics of the most useful wild plant study systems in research categories that typically include experimental manipulations of infection status and other environmental conditions. This included all studies employing wild or crop/model + wild plants within the areas of *Environmental virology, Virus effects on host traits*, and *Virus–vector interactions*. Finally, we used this analysis, and the broader analysis of precursor and product papers, to generate a set of criteria for identifying and developing new study systems.

### Host Selection, Site Selection, and Plant Sampling

Based on the quantitative literature synthesis, we evaluated California native plant species typically found in fragmented natural communities as candidate model species for basic and applied virus ecology research. We focused on perennial dicot species and identified three promising candidates: *Cucurbita foetidissima* (buffalo gourd), *Cucurbita palmata* (coyote gourd), and *Datura wrightii* (sacred thorn-apple). We characterized individual viromes of representatives of these three species collected from two sites in southern California near the Los Angeles Urban Region in Riverside County: The Motte Rimrock Reserve (University of California Natural Reserve System) and the Shipley-Skinner Multispecies Reserve (Metropolitan Water District of Southern California).

These reserves serve as prime examples of fragmented, remnant natural communities that lie adjacent to urban and agricultural systems (Figure 1). Vegetation at the Motte Rimrock Reserve (289 ha) is primarily inland coastal sage scrub. Mean annual precipitation is 33 cm; common soil types include coarse sandy loam, rocky sandy loam and sandy loam (Motte Rimrock Reserve Statistics, 2010; United States Department of Agriculture Natural Resources Conservation Service, 2017). Dominant plant families include the Lamiaceae, Asteraceae and Poaceae, with Cucurbitaceae, Solanaceae, and Cactaceae occurring in discrete, often large, populations (Motte Rimrock Species List, 2009). Vegetation at the larger Shipley-Skinner Reserve (1,012 ha) includes native inland coastal sage scrub and chaparral communities, as well as grasslands dominated by non-native species. Mean annual precipitation ranges from 25.4 to 40.64 cm throughout the reserve; common soil types include sandy loam, rocky loam, cobbly clay and rocky sandy loam (personal communication with rangers Robert Williams and Tom Ash). Dominant plant families include the Lamiaceae (particularly *Salvia mellifera* and *Salvia apiana*), Asteraceae (*Artemisia* spp.), Polygonaceae (*Eriogonum* spp.), and Poaceae (*Bromus* spp., *Avena* spp., *Stipa pulchra, Stipa cernua*, and *Melica imperfecta)* (personal communication with rangers Robert Williams and Tom Ash). As at Motte Rimrock, Cucurbitaceae and Solanaceae species are interspersed among the dominant vegetation. During the hot, dry summer months at both reserves (June–September), our target species are particularly green and apparent, as most other vegetation is senescent or dormant (Figure 1B).

All samples were collected without regard to expression of infection symptoms between 7:00 and 10:00 am over the course of several days in August 2017. At Motte Rimrock, we collected tissue from three *C. foetidissima* individuals from each of three populations 200–500 meters apart, from four *C. palmata* individuals also about 200–500 meters apart, and from two *D. wrightii* individuals at different ends of the reserve, 1,600 m distant from each other. At Shipley-Skinner Reserve, we found individuals of only two of the three species (*C. foetidissima* and *D. wrightii*). These plants were distributed unevenly and so were sampled along a north-south transect through the reserve. In total, we collected tissue from 25 plants: nine *C. foetidissima*, four *C. palmata*, and two *D. wrightii* from Motte Rimrock Reserve, and six *C. foetidissima*, and four *D. wrightii* from the Shipley-Skinner Reserve (Figure 1). We collected 10 g of leaf and stem tissue from each plant by inverting a clean plastic ziplock bag over the tissue and removing it from the plant, then sealing the bag. Bagged tissue was placed on dry ice and transported back to the laboratory within 2–3 h. Samples were carefully partitioned into 50-mL RNase-free Falcon tubes and stored at -80°C until processing.

### Extraction and Next-Generation Sequencing of Viral Nucleic Acids

In our case study, a primary goal was to illustrate how useful information could be gained by cost-effective approaches suitable for initial data collection. We emphasized NGS methods because of their capability to detect even previously unknown viruses. Numerous NGS platforms exist and new models become available quickly. We focused on a less expensive bench-top model available at a broad range of institutions: The Illumina NextSeq 500. To further reduce costs, we used the Mid Output flow cell (v2) with a paired-end 75 bp read-length. This configuration can produce up to 260M paired-end reads. For a subset of viruses, we additionally used virus-specific RT-PCR and Sanger sequencing to characterize virus infections.

Sequencing plant-infecting RNA viruses typically requires extraction of total RNA (host plus associated microbes), small RNA (21–24 nt), or double-stranded RNA (dsRNA) (Kesanakurti et al., 2016). There are advantages and disadvantages to each approach, which vary with application. If total RNA is extracted without further purification, sequencing resources will be spent on host nucleic acids, which may decrease the probability of detecting low-abundance viruses. This disadvantage can be mitigated by first conducting virion-associated nucleic acid (VANA) semi-purification (Bernardo et al., 2018) or depleting ribosomal RNA fractions. In some cases, it is effective to target small RNAs, which are often abundant in infected plants following degradation of virus genomes by conserved, anti-viral silencing mechanisms (Pooggin, 2018). However, this approach varies in feasibility with plant silencing efficiency, coverage of viral genomes may be uneven, and it is difficult to detect viral sequence variants due to the very short reads (21–24 nt). We chose to focus on extractions of dsRNA. Few plant RNA viruses have dsRNA genomes, but dsRNA forms of viral single stranded (ssRNA) genomes are present in all infected plants during the replication cycle. High molecular weight dsRNA can also be found in tissue infected with some DNA viruses (Weber et al., 2006), suggesting that dsRNA extracts can detect RNA viruses along with at least some DNA viruses, although this has not been rigorously tested. NGS of dsRNA extracts is also well suited to low-abundance viruses because dsRNA extracted from infected plants is primarily of virus origin, not host origin.

The tissue of wild plants typically is tougher than that of crop and model species and contains chemically diverse metabolite profiles, which can interfere with nucleic acid extraction and downstream molecular analysis (Lacroix et al., 2016). To evaluate the effectiveness of genomic analyses in this study, we tracked virus recovery from sampled plants by spiking each sample with a leaf punch of bell pepper cv. California Wonder containing *Bell pepper endornavirus* (BPEV, *Endornaviridae*) prior to dsRNA extraction (Kesanakurti et al., 2016). One leaf punch was added to 4 g of sample tissue contained in a 50 mL RNase-free falcon tube with 12 3-mm diameter stainless steel grinding balls. Spiked samples were homogenized using a Geno/Grinder^®^ (SPEX SamplePrep) by shaking at 1,700 rpm for 30 min (necessary for complete homogenization of 4 g of tissue). Samples were kept frozen during grinding by pre-cooling solid metal racks containing the tubes in liquid nitrogen for 5 min. We isolated double stranded RNA from 4 g of plant tissue using a low-cost extraction procedure involving binding and enrichment of dsRNA using Sigmacell cellulose type 101 powder (Tzanetakis and Martin, 2008; Kesanakurti et al., 2016). New tubes, grinding balls, and barrier tips were used throughout the extraction procedure and downstream library preparation work to prevent cross-contamination. Purified dsRNA was dissolved in 25 μl of nuclease-free water.

To construct libraries (one per sample), we denatured an aliquot of 5 μl of dsRNA solution from each extraction by incubating at 99°C for 5 min, then placed the solution on ice and used the NEBNext^®^ Ultra^TM^ II Directional RNA Library Prep Kit for Illumina^®^ by following the manufacturer’s protocol. The average insert size is 250–300 bp. We barcoded 27 libraries, which were pooled and sequenced in two separate runs (one with 12 libraries, one with 15, including two pepper controls) on an Illumina NextSeq 500 instrument using Mid Output v2 kit. We elected to use 75 bp paired end reads for cost savings. Sequencing, adapter removal and quality checks were performed by the UC Riverside genomics core facility. Sequences that could not be assigned an index based on the adapter sequence were discarded (see raw read statistics in Supplementary Data Sheet 2; adapter-removed raw reads are available in the sequence read archive of GenBank with accession number SRP149013). Reads were assembled into contigs with the Trinity assembly and analyzed with “NCBI BLAST+ blastn” using a Galaxy-based workflow (Supplementary Data Sheet 2). This workflow does not require any specialized coding skills, Linux knowledge, or data storage expenses, and thus may be more accessible to researchers new to this work. For our main analysis, we chose to subtract reads that matched the available genomes of closely related host species (*Cucurbita maxima* and *Solanum lycopersicum*). For a subset of samples, we compared this approach to a direct analysis without host genome filtering. Details of both approaches are available in Supplementary Data Sheet 2.

### Virus Identification and Phylogenetic Analysis

To identify known and novel virus sequences in our samples, we performed BlastN on the Illumina-based contigs using the “NCBI BLAST+ blastn” tool in Galaxy (Supplementary Data Sheet 2). Assemblies were compared to a previously prepared database of plant and fungal viruses^1^. We chose this database since it includes complete nucleotide sequences for plant viruses, fungal viruses, and viruses infecting both plants and fungi. To minimize false or misleading hits, we did not include partial nucleotide or amino acid virus sequences available in GenBank.

The contigs assembled included nearly complete genome sequences from four of six detected crop-associated viruses (*Tomato chlorosis virus*, [ToCV] *Closteroviridae; Cucurbit aphid-borne yellows virus*, [CABYV] *Luteoviridae*; *Zucchini yellow mosaic virus*, [ZYMV] *Potyviridae*; and *Papaya ringspot virus*, [PRSV] *Potyviridae*). We performed further phylogenetic analyses to identify possible origins and presence of host-specific variants. All sequences used for alignments and phylogenetics (Supplementary Data Sheet 3) are from the Illumina sequencing of dsRNA libraries except for ToCV, for which we amplified and Sanger-sequenced the complete coat protein coding sequence from total RNA cDNA (for sample numbers SSCF1A, SSDW4, and SSDW2B). This was necessary because our Illumina sequencing did not yield complete genomes for all samples. All other sequences used are from previously reported crop-associated virus isolates (GenBank accession numbers and geographic origins shown in Supplementary Data Sheet 4). For CABYV, PRSV, and ZYMV we selected published virus sequences based on their geographic origins (preference for adjacent geographic regions within the United States, followed by representative sequences from regions outside the United States where the virus is established). For ToCV, which was not previously known to occur in California, we constructed phylogenies using coding sequences for coat protein, minor coat protein, and RNA-dependent RNA polymerase to determine the possible origin of the ToCV detected in *D. wrightii* and *C. foetidissima*.

For analysis, we aligned coat protein coding sequences from contigs identified as representing crop-associated viruses using the freely available ClustalW tool in Mega X software (Kumar et al., 2018). Sequences in alignments were uniformly trimmed to ORF regions of interest for which we had obtained corresponding sequences from the field (Supplementary Data Sheet 3). The trimmed alignments were manually checked to verify the absence of mis-alignments, using translated amino acid sequences as a guide, and then exported to fasta format. We then conducted maximum likelihood phylogenetic analysis on each alignment in CLC Main Workbench (version 8), a low-cost, user-friendly software platform available from Qiagen. For nucleotide analyses, we first used the CLC model testing tool to determine the best substitution model for each analysis, which varied with the alignment. This tool evaluates the suitability of substitution models using four different statistical analyses, including hierarchical likelihood ratio test (hLRT), Bayesian information criterion (BIC), minimum theoretical information criterion (AIC), and the corrected minimum theoretical information criterion (AICc). The base tree for model evaluation was created by the neighbor-joining method. We selected the model with the greatest support across all four statistical evaluations. Selected models included General Time Reversible (GTR) + G + T (Yang, 1994), Kimura 80 + G + T (Kimura, 1980), and HKY + T (Hasegawa et al., 1985), where G indicates rate variation with estimated gamma distribution parameter and T indicates topology variation (see figure legends for specific selections). For amino acid analysis, we used the Whelan and Goldman (WAG) model of protein substitution (Whelan and Goldman, 2001). For the maximum likelihood phylogenetic analysis, the starting trees were created with the neighbor-joining method. One hundred bootstrap re-samples were performed for each analysis. The results are presented in unrooted radial format, drawn to scale, with branch lengths measured in the expected number of substitutions per site. Only nodes with bootstrap values ≥70 are shown; nodes with bootstrap values <70 are collapsed and not shown.

For putative novel viruses (PV1–4) we selected and aligned the longest coding sequence with a minimum of 400 bp matching the RNA-dependent RNA polymerase gene (RdRp) with corresponding RdRp coding sequences from viruses having a BlastN hit in the plant virus database. Alignments were performed using the ClustalW tool in Mega X software (Kumar et al., 2018). We chose RdRp amino acid sequences for alignments because this genome region received the most BlastN hits across samples. For *partitivirus* 1 (PV1), we excluded GenBank sequences NC003885.1 and NC004018.1 in the alignment as they were highly dissimilar to other sequences returned in the BlastN analysis. Alignment trimming, manual checks for mis-alignments, and maximum likelihood phylogenetic analysis were performed as described above for known viruses. For all analyses, we used the WAG model of protein substitution.

### Comparison of Illumina Sequencing to RT-PCR on dsRNA or Total RNA Extracts

For a subset of detected viruses (*Cucurbit aphid-borne yellows virus* [CABYV, *Luteoviridae*] and *Tomato chlorosis virus* [ToCV, *Closteroviridae*]), we performed targeted detection assays (RT-PCR) using dsRNA and total RNA isolations. Tissue used for both isolations experienced only one freeze-thaw cycle. We isolated total RNA from ∼100 mg leaf tissue using a phenol-based method (RiboZol^TM^, VWR) and dissolved extracted RNA in 100 μl of nuclease-free water. Total RNA concentrations were 100–400 ng/μl; we repeated extractions until we obtained RNA from each sample with an A260/A280 ratio of greater than 1.5. Prior work with wild plant species demonstrated that low A260/A280 ratios are associated with inhibition of reverse transcription and PCR reactions (Lacroix et al., 2016). Therefore, for reverse transcription, we used an enzyme (Superscript IV, Invitrogen) that is robust against most plant-derived inhibitors according to the manufacturer’s product description, and added RNase inhibitor (Ribolock, Thermo Fisher) to prevent RNA degradation. 5 μl total RNA was reverse transcribed, resulting in 20 μl of cDNA. We used both random hexamers (low cost, one RT reaction for all viruses) and gene-specific primers (high cost, unique RT reactions for each virus) to determine how these two approaches perform. When dsRNA extracts were processed, we first denatured the material by incubating 2 μl at 99°C for 5 min. We then placed the solution on ice and performed reverse transcription as for total RNA.

Subsequent PCR reactions (20 μl reaction volume) consisted of 1 μl of template cDNA, 4 μl of 5X HF buffer (Thermo Fisher Scientific), 1 μl of each 10 μM primer, 2 μl of dNTP mix (2 mM each) and 0.2 μl of Phusion DNA polymerase (Thermo Fisher Scientific). Primers used for PCR detection were previously developed by others (Supplementary Data Sheet 5). One PCR protocol was used for both CABYV and ToCV viruses: 98°C for 3 min for initial denaturation, followed by 40 cycles of 98°C for 10 s, 60°C for 30 s, 72°C for 1 min, then 72°C for 10 min. PCR was conducted in a Bio-Rad T100^TM^ Thermal Cycler. For Sanger sequencing of PCR products, amplicons were purified with Mag-Bind^®^ Total Pure NGS magnetic beads (Omega Bio-tek). Sanger sequencing was performed by Retrogen Inc. and primer sequences were removed from contigs prior to analysis.

## Results

### Knowledge Gaps and Resource Needs in the Field of Plant Virus Ecology

**FIGURE 2:**
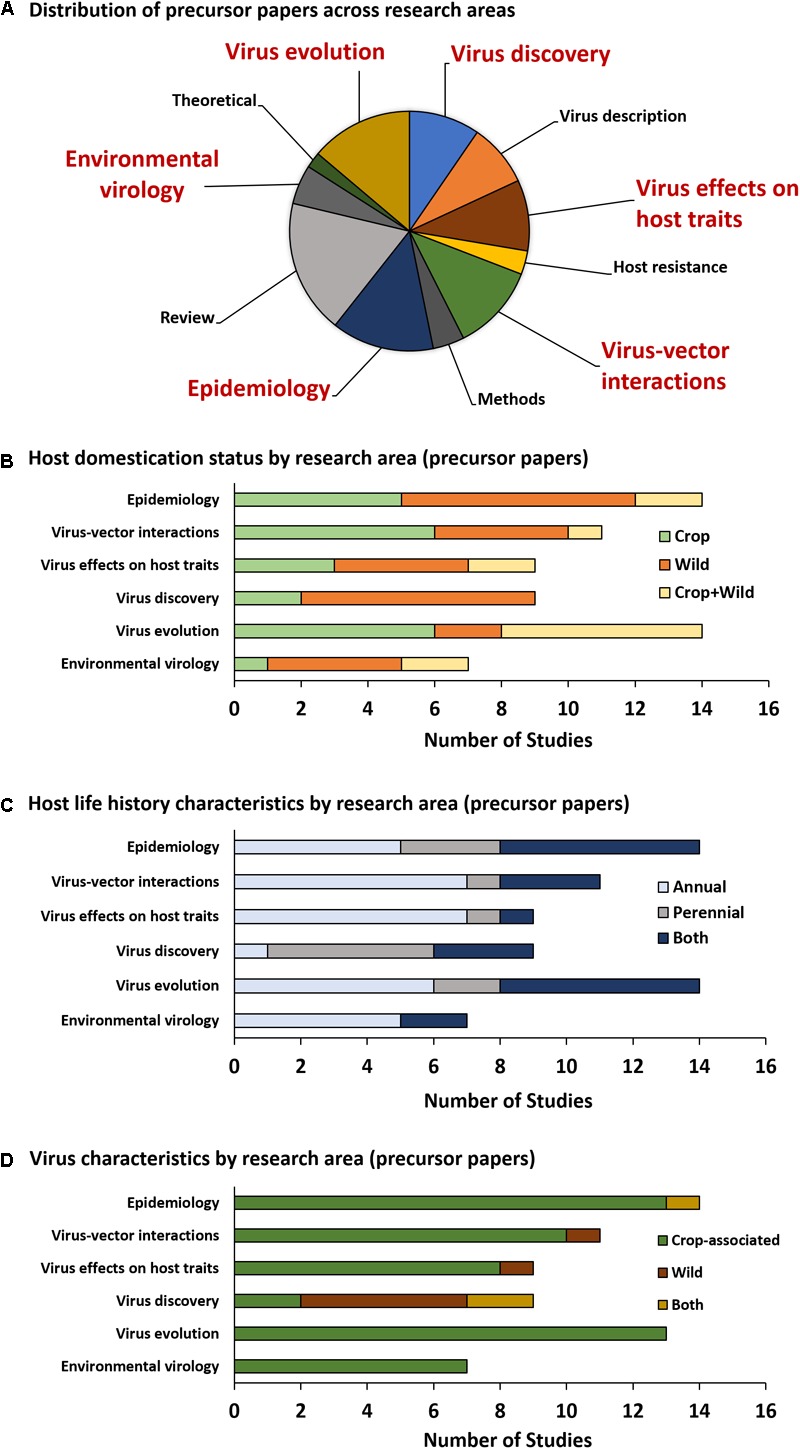
Precursor paper research topic categories. The pie graph **(A)** illustrates the proportion of precursor papers (direct outputs of PVEN) falling into each category. The six categories most relevant to the field of virus ecology are emphasized in large, red text. Graphs **(B–D)** summarize the number of studies utilizing hosts and viruses with specific categorical characteristics.

Among precursor papers (Figure 2A), studies focusing exclusively or partially on wild plant hosts have equal or greater representation relative to studies focusing exclusively on crop hosts within the areas of *Environmental virology, Epidemiology, Virus effects on host traits, Virus evolution*, and *Virus discovery*, while studies focusing exclusively or partially on wild hosts are somewhat underrepresented within the area of *Virus–vector interactions* (5/11 studies) (Figure 2B). Studies focusing exclusively on annual hosts are overrepresented in the areas of *Environmental virology* (5/7 studies, with none on perennials alone), *Virus effects on host traits* (7/9 studies), and *Virus–vector interactions* (7/11 studies) (Figure 2C). *Virus discovery* is the only research area in which studies focusing exclusively on perennial hosts (5/9) outnumber those focusing exclusively on annual hosts (1/9) (Figure 2C). Nearly all studies, regardless of research area, focus on crop-associated viruses, except for the *Virus discovery* category, in which 7/9 studies included wild viruses that are not causative agents of disease in crops (Figure 2D).

**FIGURE 3:**
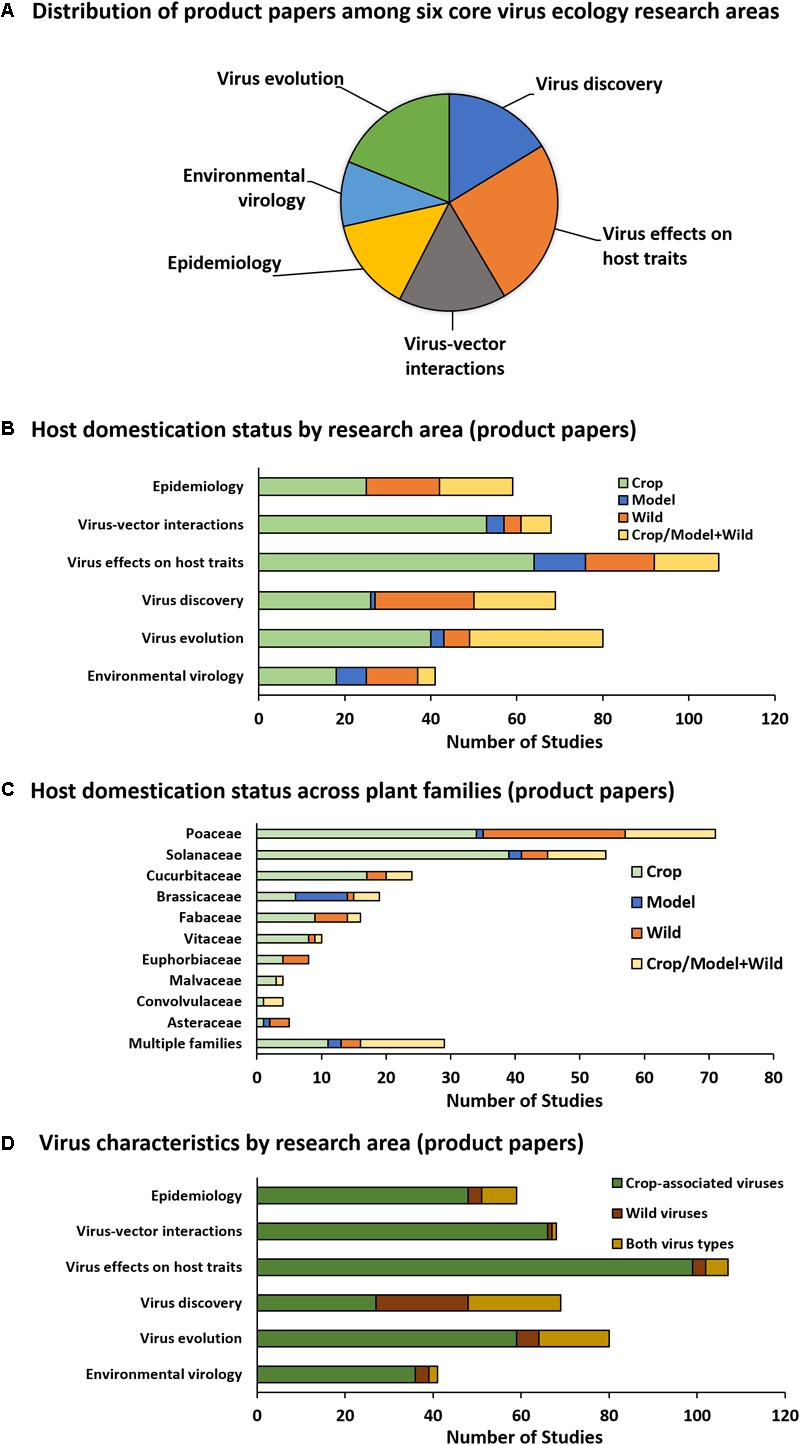
Product paper analysis. The pie graph **(A)** illustrates the proportion of product papers (those citing direct outputs of PVEN) falling into each core plant virus ecology research area. Graph **(B)** illustrates the domestication status of hosts studied in product papers organized by research area. Graph **(C)** illustrates domestication status of hosts studied in product papers organized by plant family and pooled across research areas. Families depicted are those represented by >3 studies. Families not depicted include Actinidiaceae (one study, crop), Amaranthaceae (one study, crop), Amaryllidaceae (two studies, crop/model + wild, one study, wild), Apocynaceae (one study, wild), Orchidaceae (one study, crop/model + wild, one study, wild), Rutaceae (three studies, one each in crop, wild, and crop/model+wild), and Zingiberaceae (one study, crop). Graph **(D)** illustrates virus characteristics organized by research area.

Among product papers falling into one or more of the six core virus ecology research areas (Figure 3A), there is a clear shift toward greater proportional representation of crop and model host plants relative to wild hosts (Figure 3B). When each exclusive category (crop, model, or wild) is considered alongside the combined category, studies employing wild hosts only enjoy equal representation with crops or models in the *Virus discovery* and *Epidemiology* categories – both of which are heavily based on surveys rather than manipulative studies. In contrast, studies on wild hosts are lacking in areas that typically employ experimental manipulations (*Virus effects on host traits, Virus–vector interactions*, and *Virus evolution*) (Figure 3B). When host domestication status is examined along an axis of plant family rather than research category, additional biases are apparent (Figure 3C). Nearly half of all studies focusing exclusively on wild plants, and about one-third of studies that include both wild and cultivated plants, were performed with monocots from a single plant family (Poaceae) (Figure 3C). Solanaceous hosts are also popular, but only a quarter of studies on Solanaceae include wild species (Figure 3C). About 80% of studies focused exclusively on crop-associated viruses, with only the *Virus discovery* research area (which is primarily based on survey work) including wild viruses in about 60% of studies (Figure 3D).

**FIGURE 4:**
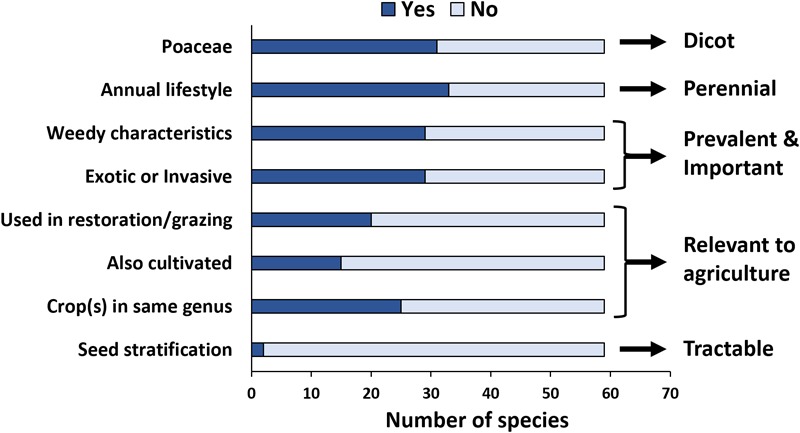
Characteristics of wild hosts used as focal research organisms in experimental virus ecology studies. In total, 59 wild plant species were studied across all product papers in three research areas that frequently involve factorial or manipulative empirical studies (*Virus–vector interactions, Virus effects on host traits*, and *Environmental virology*). Arrows and text to the right indicate desirable characteristics of potential model hosts for experimental approaches.

Among product papers within the areas of *Virus effects on host traits, Virus–vector interactions*, and/or *Environmental virology* that included wild hosts, we detected overrepresentation of monocots (specifically, Poaceae), overrepresentation of annuals (most wild plant communities are dominated by perennials), a preference for uncomplicated seed-based propagation methods, and a tendency to prefer exotic/invasive hosts with weedy characteristics (Figure 4). Despite the fact that only a fraction of wild plants are ancestral to crops, more than one-third of wild hosts studied are in the same genus as a crop species, and about one quarter to one-third are themselves cultivated in some context or selected for use in landscape manipulation (restoration or generation of fodder for grazing by ruminants) (Figure 4).

### Virus Detections in Model Wild Dicot Hosts: Illumina Sequencing

**FIGURE 5:**
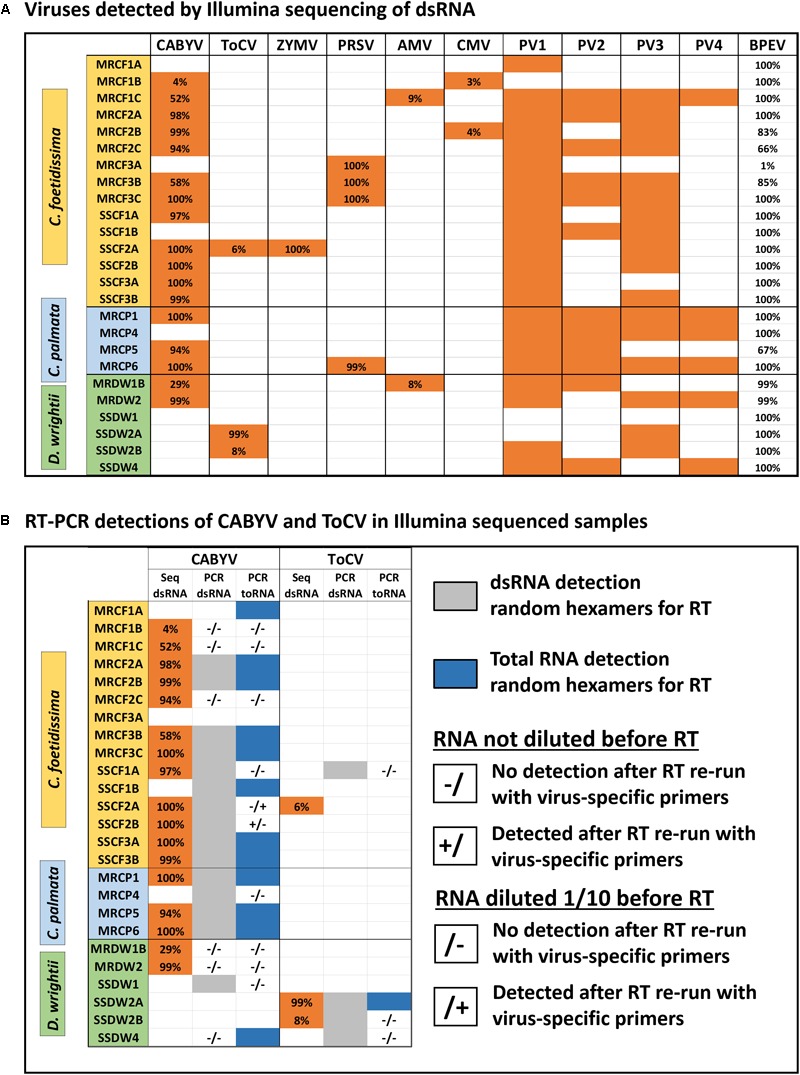
Viromes of individual plants **(A)** and summary of detections by different diagnostic methods for select crop-associated viruses **(B)**. Sample codes within each host plant section indicate species (CF, CP, or DW), site of collection (MR, Motte Rimrock; SS, Shipley-Skinner), numbers (1, 2, and 3) indicate populations within a site, and letters (A, B, and C) indicate individuals within each population. Viruses are as follows, from left to right: *Cucurbit aphid-borne yellows virus, Tomato chlorosis virus, Zucchini yellow mosaic virus, Papaya ringspot virus, Alfalfa mosaic virus, and Cucumber mosaic virus.* PV1–4 refer to novel viruses related to members of the family *Partitiviridae*. The last column shows the coverage of the internal control (*Bell pepper endornavirus*) for each sample. Results are from assemblies constructed after subtraction of host genomes (see the section “Materials and Methods”). We found no effect of host subtraction on the identity and number of viruses detected (see the section “Evaluating Detection Biases in Bioinformatic Workflows and Downstream Applications” in the Discussion).

**FIGURE 6:**
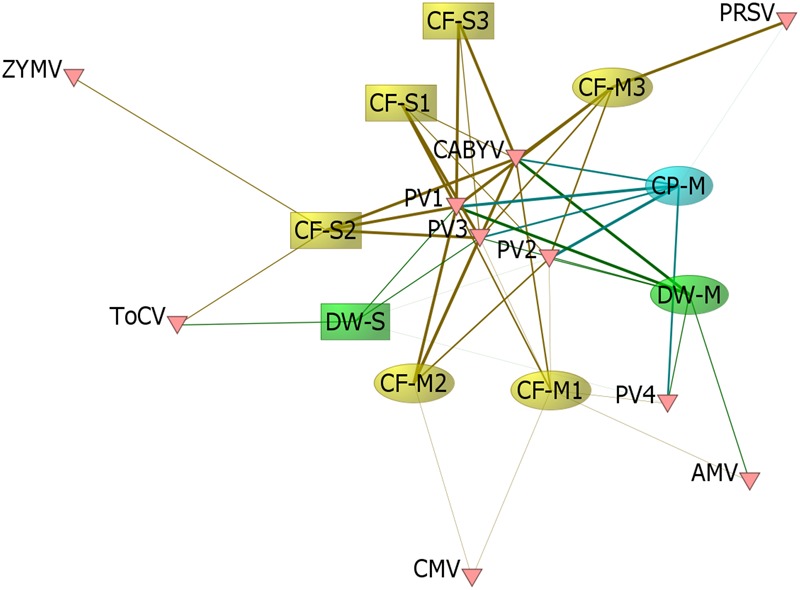
Viral links among hosts. We constructed a matrix of the 9 population groups sampled × 10 viral groups detected, where values for each population within the matrix represented the proportion of individuals sampled in which Illumina sequencing detected the corresponding virus group. We visualized the network structure with the network analysis software ORA-Lite version 11 (Carley et al., 2018), where the network was sized by betweenness centrality. The width of the edge between a given population and virus group represents the proportion of individuals with Illumina-detected infection. Sample codes indicate species (CF, CP, or DW), site of collection (M, Motte Rimrock; S, Shipley-Skinner), and population number within a site (1, 2, and 3).

We detected six crop-associated viruses via dsRNA extraction and Illumina sequencing followed by host genome filtering (Figure 5A and Supplementary Data Sheet 6). For 65% of these detections we recovered greater than 90% of the virus genome. All of the detected crop-associated viruses are known to be present in California, with the exception of ToCV, a whitefly transmitted virus that is established in the Southeastern United States (Wintermantel and Wisler, 2006). We confirmed infections by ToCV in at least two *D. wrightii* plants via RT-PCR and Sanger sequencing, all of them from the Shipley-Skinner reserve site. We also identified one instance of ToCV infection in a cucurbit host (*C. foetidissima*) growing in the same site as *D. wrightii* plants infected with ToCV (Figures 1, 5). Although it is primarily a pathogen of cucurbits, we also detected CABYV infections in *D. wrightii* (Figure 5A). In addition to crop-associated viruses, we detected several viruses most closely related to members of the *Partitiviridae*, which we partitioned into four groups (PV1–4) based on sequence similarity among isolates (Figure 5A). Most of the plants we sampled harbored viruses in groups PV1–3, while viruses in group PV4 were less frequently detected. A network analysis based on Illumina detections (Figure 6) illustrates the number and strength of interactions among viral players across host species and sampling locations. This analysis suggests that one crop-associated virus, CABYV, is strongly associated with both cucurbit species, with potential transmission to the solanaceous host. The other crop-associated viruses are peripheral, appearing only sporadically within select populations. The putative *Partitiviridae* within each of the PV1–3 groups also have strong connections with all hosts, and are present in both sampling sites, while viruses in the PV4 group occupy an intermediate position between the center and the periphery.

### Detection Efficiency for Crop-Associated Viruses

The internal control of pepper tissue infected with BPEV worked well as a metric to assess dsRNA recovery and sequencing depth. In most samples, we recovered the entire genome of BPEV. But in one case, we recovered only 1% of the genome (sample MRCF3A – Figure 5A). This sample was left within our data set to illustrate how recovery of the BPEV genome tracks with detection of other viruses. Sample MRCF3A had fewer co-infections than most other samples based on Illumina sequencing. RT-PCR was performed to amplify a 381 bp fragment of BPEV in the pepper-spiked dsRNA extraction from this individual (Supplementary Data Sheet 5). The fragment was detected, suggesting that dsRNA isolation was successful, but possibly compromised in a way that lowered abundance of virus dsRNA below the detection threshold for sequencing. Use of BPEV as an internal control enables attribution of the source of this variation to processing errors rather than biological factors.

Wild plants often contain inhibitors of RT and PCR reactions (Lacroix et al., 2016). If our candidates are particularly recalcitrant for targeted detection assays, this will alter their suitability as models for virus ecology work and/or require additional method optimization. For CABYV and ToCV, we found good congruence between detection via Illumina sequencing and detection via RT-PCR using random hexamers during the RT reactions for both dsRNA and total RNA (Figure 5B). Additionally, two samples tested positive for ToCV and five samples tested positive for CABYV without testing positive via Illumina sequencing (Figure 5B). For a subset of samples with discrepancies among the three detection methods, we repeated the protocol with virus-specific primers, and with or without RNA dilution (Figure 5B) (Lacroix et al., 2016). Detection was only improved for two samples: SSCF2A, where CABYV was detected in the 1/10 dilution with specific primers, and SSCF2B, where CABYV was detected in the undiluted sample with specific primers.

### Phylogenetic Analyses of Crop-Associated and Novel Viruses

**FIGURE 7:**
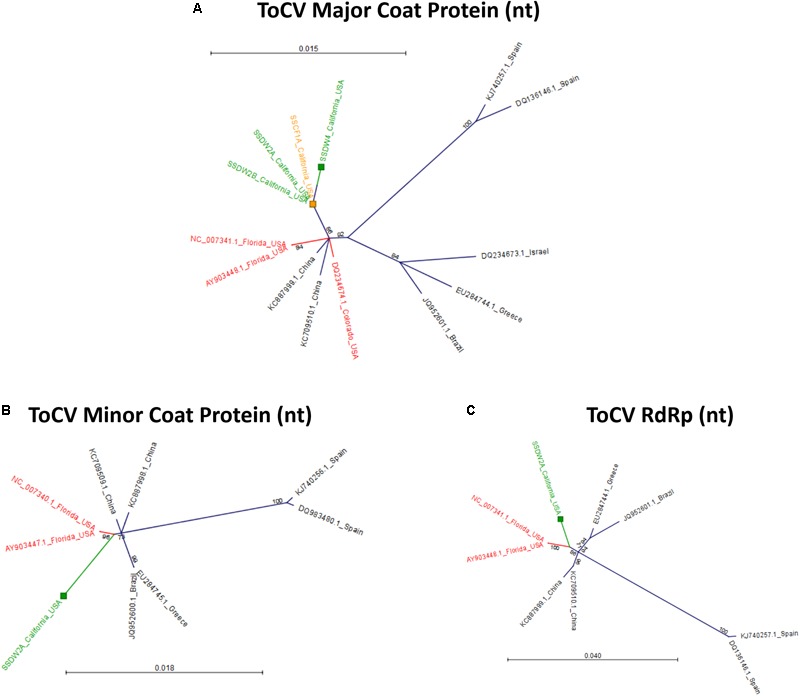
Radial, unrooted trees illustrating results of the maximum likelihood phylogenetic analyses of ToCV nucleotide sequences. For the major coat protein **(A)**, the HKY + T model was used. For the minor coat protein **(B)**, the GTR + G + T model was used. For the RNA-dependent RNA polymerase **(C)**, the HKY + T model was used. Color coding has been applied to aid interpretation of sequence origins (green = *D. wrightii*, orange = *C. foetidissima*, red = other sequences from United States isolates). One hundred bootstrap re-samples were performed for each analysis. Branch lengths are measured in the expected number of substitutions per site. Only nodes with bootstrap values ≥70 are shown; nodes with bootstrap values <70 are collapsed and not shown.

**FIGURE 8:**
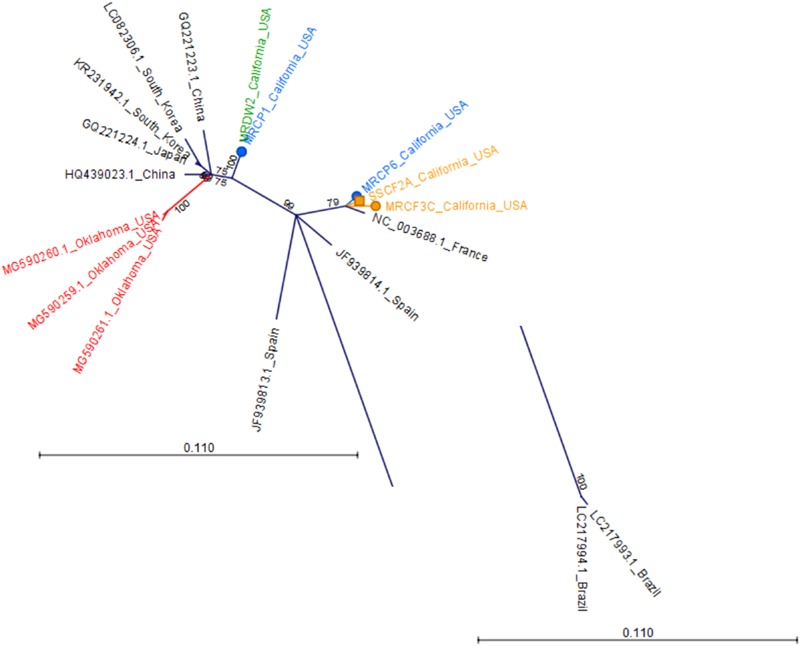
Radial, unrooted trees illustrating results of the maximum likelihood phylogenetic analyses of CABYV coat protein nucleotide sequences. The GTR + G + T model was used for tree construction. Two Brazilian CABYV isolates exhibit extreme divergence from all other sequences and the branch containing these isolates has been trimmed to improve readability of the tree. Color coding has been applied to aid interpretation of sequence origins (green = *D. wrightii*, orange = *C. foetidissima*, blue = *C. palmata*, red = other sequences from United States isolates). One hundred bootstrap re-samples were performed for each analysis. Branch lengths are measured in the expected number of substitutions per site. Only nodes with bootstrap values ≥70 are shown; nodes with bootstrap values <70 are collapsed and not shown.

**FIGURE 9:**
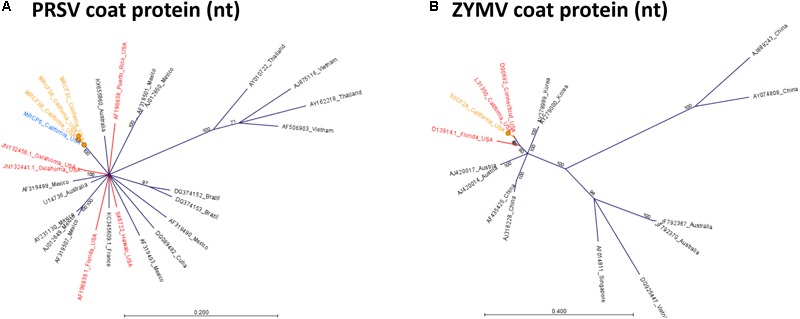
Radial, unrooted trees illustrating results of the maximum likelihood phylogenetic analyses of *Potyvirus* nucleotide sequences. For PRSV **(A)**, and ZYMV **(B)** the GTR + G + T model was used. Color coding has been applied to aid interpretation of sequence origins (orange = *C. foetidissima*, blue = *C. palmata*, red = other sequences from United States isolates). One hundred bootstrap re-samples were performed for each analysis. Branch lengths are measured in the expected number of substitutions per site. Only nodes with bootstrap values ≥70 are shown; nodes with bootstrap values <70 are collapsed and not shown.

To determine relationships among virus isolates and explore geographic origins, we performed a phylogenetic analysis for all crop-associated viruses for which we recovered all or most of the genome (ToCV, CABYV, PRSV and ZYMV). ToCV is not known to be present in California and our detection represents the first instance of this virus being present in this state. To determine possible ToCV origins, we used sequences on GenBank for the major and minor coat proteins and the RNA-dependent RNA-polymerase. All trees suggest that our ToCV sequences are most similar to those of isolates collected within the continental United States (Florida and Colorado), and to those of isolates collected in China (Figure 7). There is no evidence that the ToCV infecting *C. foetidissima* is distinct from ToCV infecting *D. wrightii* hosts in the same reserve site (Figure 7A). Phylogenetic analysis of the CABYV coat protein sequence suggests the presence of two genotypes (Figure 8). The first CABYV genotype is most similar to isolates collected in Asia (China, Japan, Korea) and the midwestern United States (Oklahoma). This genotype infects both *C. palmata* and the solanaceous host, *D. wrightii*. The second CABYV genotype groups with isolates from Europe, and we found no evidence of this genotype infecting *D. wrightii*. Phylogenetic analysis of our PRSV isolates suggests a single genotype present in the Motte Rimrock Reserve site (Figure 9A). Origins are not clear, as our PRSV group is equivalently similar to isolates from the United States, Mexico, and Australia (Figure 9A). In contrast, based on the coat protein sequence, our ZYMV isolate is likely of United States origin, with the most similar sequence being from another California isolate collected from an agricultural field (Figure 9B).

**FIGURE 10:**
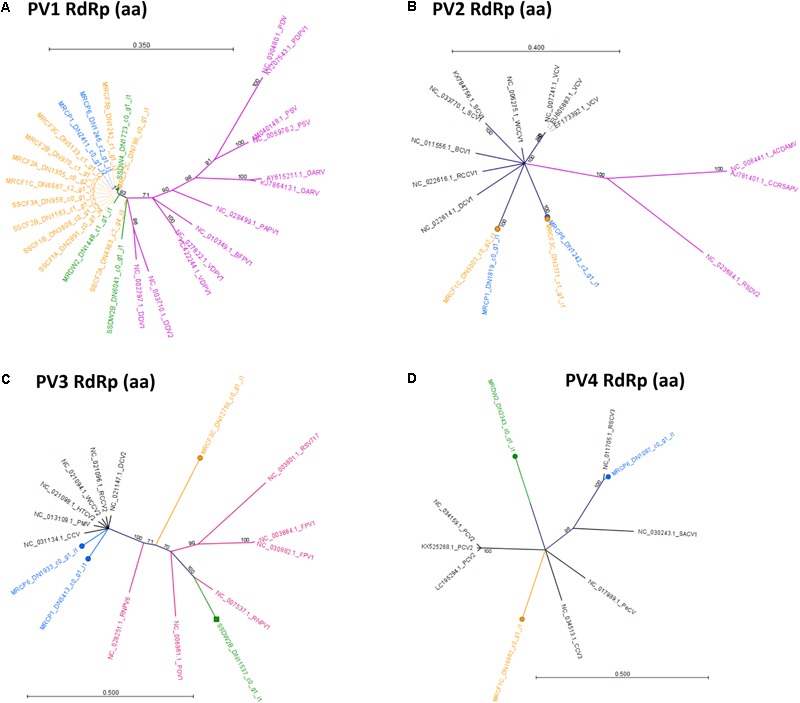
Radial, unrooted trees illustrating results of the maximum likelihood phylogenetic analyses of *Partitiviridae* amino acid sequences. Trees in **(A–D)** illustrate relationships among putative *Partitiviridae* detections reported in Figure 5. Color coding has been applied to aid interpretation of sequence origins (Trinity assemblies → green = *D. wrightii*, orange = *C. foetidissima*, blue = *C. palmata*; GenBank sequences → purple = fungus-infecting viruses, black = plant-infecting viruses). Full virus names and genera for GenBank sequences are available in Supplementary Data Sheet 8. One hundred bootstrap re-samples were performed for each analysis. Branch lengths are measured in the expected number of substitutions per site. Only nodes with bootstrap values ≥70 are shown; nodes with bootstrap values <70 are collapsed and not shown.

Phylogenetic analysis of RdRp amino acid sequences from detected *Partitiviridae-*like viruses revealed two possible virus lifestyles, most of which are consistent with patterns of detection among our three hosts and sampling sites. The PV1 group appears to contain sequences from a single virus species, as the sequences detected shared 100% identity. The PV1 group drew BlastN hits only for fungus-infecting *Partitiviridae* in the genus *Gammapartitivirus*, which does not include any plant-infecting viruses (Figure 10A). A fungus-infecting lifestyle is consistent with the finding that PV1 is frequently present in both cucurbits and *D. wrightii* hosts across two sites (Figure 5A). Plants have numerous fungal endophytes and other associates. PV1 viruses could infect a generalist fungus associate of all three hosts, but this requires additional validation. The PV2 group contains at least two virus genotypes, which possibly represent two species, as the distance between the two clades is greater than that between different characterized viruses included in the same tree (Figure 10B). Members of each group are present in both *C. foetidissima* and *C. palmata* tissues. The PV2 group members are most similar to plant-infecting members of the genus *Alphapartitivirus*, with fungus-infecting members of this genus being slightly more distant in branch length. However, PV2 sequences were also detected in *D. wrightii*, although a large enough sequence was not available from *D. wrightii* to include in this analysis (Figure 5A). Based on this, a mycovirus lifestyle should not be ruled out. Analysis of PV3 sequences also suggests that this group may contain multiple species with different lifestyles. *C. palmata* derived sequences group closely with plant-infecting *betapartitiviruses*, while the isolates from *C. foetidissima* and *D. wrightii* group with fungus-infecting *betapartitiviruses* (Figure 10C). In contrast, group PV4 appears to contain three distinct *Partitiviridae-*like viruses that are most similar to plant-infecting species (Figure 10D). The only classified member in this tree is PCV2 (*Pepper cryptic virus 2*, genus *Deltapartitivirus*).

## Discussion

### Knowledge Gaps and Resource Needs in the Field of Plant Virus Ecology

Plant virus ecology is an expanding discipline. Its origins are rooted in the managed plant systems that served as the basis for advancements in plant virology, but its strengths lie in the translation of this research to ecological questions, and model systems, outside of agriculture. To quantify its development, we evaluated outputs of the NSF-funded PVEN, which ignited the field of plant virus ecology by bringing together research teams and propagating new syntheses (NSF Award Abstract #0639139, 2011). We further quantified the impact of these outputs (precursor papers) by evaluating the ecological scope of studies that built upon them (product papers). Our quantitative synthesis of precursor papers suggests that the efforts of PVEN succeeded in promoting studies on virus interactions with non-cultivated (wild) host plants (Figure 2A); most research categories had equal or greater representation of wild hosts relative to crop hosts. Although the majority of hosts were annuals, a sizeable proportion of studies included perennials as well (Figure 2C). And within the *Virus discovery* research area, several studies focused on detecting and describing viruses not associated with disease in crops (Figure 2D), which could serve as the focus of future studies in research areas outside of *Virus discovery*.

These patterns reflect a desire and need to expand outside of agricultural systems while maintaining study system feasibility. Annual wild plants are often more suitable for manipulative experiments than perennials due to faster generation times and greater greenhouse tractability. Crop-associated viruses are readily available as pure isolates and research on these viruses is fundable from both basic and applied grant sources. It is logical that initial efforts to expand the field of virus ecology would do so by hybridizing features of agricultural systems with wild systems. However, our analysis of product papers suggests that this hybrid approach is becoming less common and there is a reversion to reliance on crop systems for addressing ecological questions. Among product papers, the only research areas having equitable proportional representation of wild plants with crops or models (either alone or in combination) are those in which collection surveys dominate over experimental approaches (*Epidemiology, Virus discovery*, and *Virus evolution*) (Figure 3B). More troubling, crops and laboratory models (e.g., *Nicotiana benthamiana*) are preferred as hosts in the core research areas that are most likely to use manipulative experimental approaches to understand the roles of viruses in plant ecology (*Virus–vector interactions, Virus effects on host traits*, and *Environmental virology*) (Figure 3B). Within these studies, wild plant hosts are not diverse—about half of all studies on wild plants use hosts from a single family: the Poaceae (Figures 3C, 4).

Our quantitative synthesis suggests that there are persistent trade-offs in virus ecology research based largely on pathosystem logistics. This assessment is supported by our more detailed analysis of wild hosts used in studies within the *Virus effects on host traits, Virus–vector interactions*, and *Environmental virology* research areas (Figure 4). Researchers prefer annuals, hosts that are common and prevalent (e.g., those exhibiting weedy characteristics or invasive tendencies), hosts that are related to crops or marginally domesticated, and hosts with easy propagation (Figure 4). As a result of these biases in host choice, and a preference for using crop-associated viruses in manipulative studies, there are, and will continue to be, lingering gaps in our understanding of the ecological roles of plant viruses in unmanaged systems. But these selections are not driven by neglect, but rather the need to learn what we can from what we have available in a time frame that aligns with funding sources. Altering this entrenched framework is not feasible for a single discipline. However, our quantitative synthesis reveals several avenues for enhancing the breadth of tractable study systems available for virus ecology research given these constraints. For example, we found that there is a pressing need for more studies on dicot perennials. We also found that researchers prefer to use hosts that are easy to find in the environment, relevant to agriculture, and simple to grow and propagate in the greenhouse. Therefore, the plant virus ecology research community should be working to identify and develop new wild dicot perennial systems that meet these criteria (Figure 4). This goal formed the basis of our case study to characterize virus infections in several native, perennial dicots that are keystone species within an important Mediterranean-climate biodiversity hotspot—the Southern California Floristic Province (Myers et al., 2000).

### Viruses Detected via Illumina Sequencing of dsRNA From Candidate Model Hosts

Based on the criteria developed from our quantitative synthesis, we selected three co-occurring perennials, *C. foetidissima, C. palmata*, and *D. wrightii*, as candidate model systems. All three hosts satisfy the criteria laid out in Figure 4. The two cucurbits are prevalent in Mediterranean-climate plant communities within California and in other arid grassland/shrub communities throughout the United States. They are in the same genus as important crop hosts (summer and winter squash, gourds, and pumpkins), which have long served as models for understanding chemically mediated interactions among plants, microbes, and insects in agroecosystems (Mauck and Shapiro, 2018). *Datura wrightii*, while not as closely related to crops, is one of the few wild perennial plants developed as a model for field-based chemical ecology research (van Dam and Hare, 1998; Hare and Sun, 2011). Expanding use of this model to include plant virus ecology is a natural extension of this research.

**FIGURE 11:**
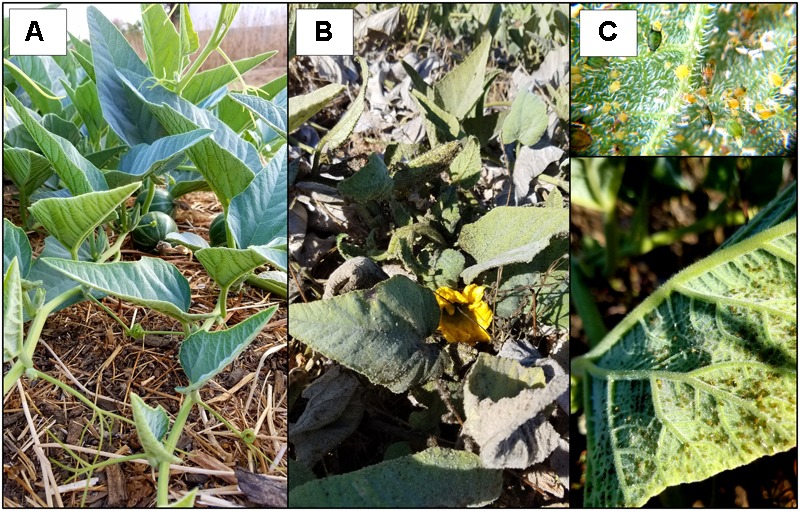
Effect of *Aphis gossypii* feeding on *C. foetidissima.* **(A)** Uninfested mature *C. foetidissima* plant. **(B)** *C. foetidissima* with heavy infestation by *A. gossypii.* Leaves are covered in sooty mold and exhibit premature senescence. **(C)** Typical *A. gossypii* densities on *C. foetidissima* leaves during heavy infestations.

Using low-cost Illumina sequencing, basic molecular techniques, and open-source bioinformatics tools, we demonstrated that our candidate model hosts frequently harbor infections by viruses common in agricultural crops, as well as crop-associated viruses not known to be present in the region (ToCV). We also found evidence of novel host family associations (CABYV in *D. wrightii* and ToCV in *C. foetidissima*) (Figure 5). Along with crop-associated virus detections, we uncovered multiple plant-associated viruses that appear related to known members of the family *Partitiviridae*, several of which are most similar to vertically transmitted plant-infecting viruses (Figures 5A, 10D). Our network analysis reveals the importance of certain viruses in the sampled host populations, as well as the ways in which hosts are connected by their virus associates (Figure 6). Surprisingly, most crop-associated viruses are peripheral within the network. Because observational studies such as ours focus on the residual plants that have persisted in the face of infection pressure, further ecological studies are needed to determine the causes of this pattern. It may indicate low levels of infection pressure, strong control of infection by hosts, or high mortality among infected hosts. The interesting exception is CABYV, which has strong connections to cucurbit hosts at both sampling sites and may be adapting to infect co-occurring *Datura* hosts. CABYV is transmitted in a persistent circulative manner by several aphid species, with the most efficient vector being the generalist cotton-melon aphid, *Aphis gossypii*, a global crop pest and vector of many viral plant pathogens. During subsequent field work in the 2018 season, we observed that *A. gossypii* is abundant on both cucurbit hosts. Populations build over the summer and peak in late August to early September. Density on individual hosts can become so high that tissue necrosis and premature senescence occur (Figure 11). Based on these observations, and our finding that CABYV is prevalent across both sampling sites, we hypothesize that this virus–vector association is a significant part of the ecology of both cucurbit hosts. This virus may modify host traits, such as growth rates and flowering patterns (Alexander et al., 2017), or tolerance of abiotic stress (Xu et al., 2008; Westwood et al., 2013; Davis et al., 2015) in ways that alter survival and multi-year fitness. CABYV can also be transmitted by *Myzus persicae*, another generalist vector that can feed on both cucurbitaceous and solanaceous crop hosts (UC Statewide IPM Program, 2016). We observed *M. persicae* in low numbers on *Datura* and the invasive tree tobacco, *Nicotiana glauca*, and we hypothesize that this vector may be responsible for CABYV transmission from perennial cucurbits to *D. wrightii.* Using tissue from our CABYV positive *C. foetidissima* and laboratory-reared *A. gossypii*, we recently transmitted this pathogen to cultivated melons and future work will experimentally test host associations detected via Illumina sequencing.

### Ecological Insights From Phylogenetic Analyses of Crop-Associated Viruses

ToCV infections in *D. wrightii* and *C. foetidissima* confirm that this virus is present in California, even if it is not currently reported infecting solanaceous crops. Our ToCV isolates group together in the coat protein nucleotide sequence tree, with good bootstrap support for divergence from other United States isolates (Figure 7A). And phylogenies based on the minor coat protein and RdRp sequences both indicate that the closest related isolates from Florida are still distinct from the ToCV detected in our study (Figures 7B,C). However, more sequence data and host range studies are needed to assess whether our California ToCV isolates represent novel variants circulating in wild plant populations and not recent introductions from crops. ToCV is present in Mexico within greenhouse production of solanaceous crop seedlings and illegally imported plant material from this region is one potential source of the virus. At present, there are no publicly available coding sequences for the coat protein, minor coat protein, or RdRp of Mexican ToCV isolates, but our results suggest that greater sampling in this region would help to clarify potential pathways for ToCV introduction to California.

There is no evidence that the ToCV isolate from the novel host (*C. foetidissima*) has diverged from isolates infecting solanaceous hosts (Figure 7A). The coat protein nucleotide sequence of the ToCV isolate from *C. foetidissima* (SSCF1A) is not distinct from sequences of two other *D. wrightii* isolates collected at the same site (SSDW2B and SSDW2A). Deep sequencing also revealed instances of CABYV infecting *D. wrightii* (Figure 5A). Based on studies with crops, this virus is known to infect numerous cucurbit hosts and a few diagnostic species, but failed to infect crops in the Solanaceae in host range studies (Lecoq et al., 1992). Again, there is no evidence that the CABYV infecting the novel host is distinct from CABYV infecting a host that is related to known susceptible crop species (*C. palmata*), although two groups of CABYV are present in our populations, and only one contains an instance of infection in *D. wrightii* (Figure 8). Additional targeted sampling of isolates from a larger number of hosts within our sites is necessary to determine if more host-specific genotypes co-occur alongside those capable of infecting hosts across the Cucurbitaceae and Solanaceae.

Even with limited sampling, our results demonstrate that virome characterization in wild perennial hosts can serve to expand our understanding of crop-associated virus host ranges while monitoring for new pathogens. Unusual associations might not occur in short-lived annual systems, but perennial hosts could undergo repeated exposure to inoculum from infected heterospecific perennial neighbors or adjacent agricultural crops over successive seasons. At present, we lack information on the effects of repeated inoculum exposures in perennials, and whether these lead to more virus diversity and mixing than in annuals. We might also expect that infection by one virus could modify susceptibility to subsequent pathogen inoculation, allowing novel associations to occur (Mukasa et al., 2006; Syller, 2012). For example, one cucurbit host infected with ToCV (genus *Crinivirus*) was also infected with ZYMV (Genus *Potyvirus*). Synergistic interactions among criniviruses and potyviruses have been documented in crop systems (Mukasa et al., 2006) and are one mechanism by which viruses overcome resistance traits. Additionally, while the perennial host genotype remains constant, the virus genotype will vary, creating opportunities for propagation and persistence of novel genotypes in new hosts if compatible insect vectors are present. In our pathosystems, *C. foetidissima* and *D. wrightii* are both highly suitable wild hosts for a major ToCV vector (*Bemisia tabaci* MEAM1) (van Dam and Hare, 1998; Wintermantel et al., 2016) and, as discussed in section 4.2, CABYV is transmitted by generalist aphids that feed on both cucurbit and solanaceous hosts. *C. foetidissima* and *D. wrightii* are also some of the only plants that are actively growing in our sites during the arid summer months, making them targets for vector activity in the absence of other options.

### Descriptions of Novel Viruses

The “wild” viruses we detected are all putative members of the family *Partitiviridae*, which includes fungal, plant, and protozoan viruses (Nibert et al., 2014). PV1 sequences are all closely related and are most similar to fungus-infecting members of the genus *Gammapartitivirus*. In our survey, nearly identical PV1 sequences were associated with all three host plant species (Figures 5A, 10A), suggesting that this virus could be infecting one or more generalist fungi capable of living in or on leaf tissue of diverse hosts. Groups PV2–PV3 contain several possible *Partitiviridae* species, some of which group most closely with mycoviruses (Figures 10B,C). Complete characterization of putative mycoviruses is beyond the scope of our study and will require isolation of individual fungal associates from plant tissue and subsequent sequencing of the viromes of these pure isolates (Ong et al., 2017). Given that fungal viruses are also known to modify the outcomes of fungus–plant associations (Márquez et al., 2007), a deeper exploration of the viromes of plant-associated fungi could be of equal importance to expanded efforts to understand plant virus diversity and prevalence in more host species.

Unfortunately, far less is known about the ecological roles of plant-infecting *Partitiviridae* despite growing evidence that such viruses are common in wild plants (Wren et al., 2006; Roossinck, 2014; Thapa et al., 2015). Our study is no exception, with all three plant species hosting at least one possible plant-infecting member of the *Partitiviridae* (sequence group PV4 – Figure 10D). Plant-infecting *Partitiviridae* are dsRNA viruses transmitted vertically only during host reproduction. They are not transmissible via grafting, mechanical inoculation, or vectors (Valverde and Navas-Castillo, 2013). Because of the difficulty in obtaining genetically identical virus-free lines, little information is available about the impact of exclusively vertically transmitted viruses on wild plant hosts.

In a subset of the crop systems for which virus-infected and virus-free lines are available, significant changes in phenotype and disease resistance due to infection with vertically transmitted viruses are evident (Valverde and Navas-Castillo, 2013). Performing such studies in wild systems will be challenging, as vertical transmission is nearly 100%. However, our prevalence data for the most well-sampled species (*C. foetidissima*) (Figure 5A) indicates that not all individuals are infected with putative plant-infecting *Partitiviridae* (PV4), even within a population. Future studies will leverage this natural variation, tractability of *C. foetidissima* for greenhouse research, and genomic resources for the genus *Cucurbita* (Paris, 2017; Sun et al., 2017), to begin exploring the biological relevance of dsRNA virus infections for plant fitness, stress tolerance, and resistance to infection by insect-vectored viruses.

### Evaluating Detection Biases in Bioinformatic Workflows and Downstream Applications

We devised and tested a virome profiling workflow consisting of an inexpensive dsRNA extraction protocol (Kesanakurti et al., 2016), economical and rapid Illumina mid-output 75 bp paired-end sequencing using the NextSeq 500 platform, and a user-friendly Galaxy-based bioinformatics protocol for virus discovery. This approach worked well in the context of our study, the goal of which was detection of known crop-associated viruses and initial discovery of putatively novel viruses. But we acknowledge that longer reads are preferred for these purposes. New technologies with the capability to produce long reads, such as the Nanopore platforms, are being validated as tools for virus discovery, and we expect these tools will become popular for plant virus ecology work. To explore the efficacy of our approach as a tool for enabling researchers from non-virology disciplines to perform virus discovery and prevalence studies with wild hosts, we compared options for pre-assembly host genome filtering and performed RT-PCR assays using previously published primer sets. Pre-assembly host genome sequence subtraction is often included as a step in virus discovery workflows to reduce the computational load during assembly (Daly et al., 2015). However, if this process removes virus sequences, it could hinder rather than help downstream analyses. In our study, read filtering against a host genome in the same genus (*Cucurbita maxima*) or subfamily (*Solanum lycopersicum*) significantly reduced the number of reads going into the BlastN analysis for both cucurbits (up to 83% of reads subtracted), but had little effect when applied to the more distantly related *D. wrightii* host (up to 14% of reads subtracted) (Supplementary Data Sheet 7). Meanwhile, the identity of detected viruses and number of detections did not change (Supplementary Data Sheets 6, 7). Our comparison suggests that a significant proportion of reads are host-derived, even when dsRNA is the extraction material, but sequence subtraction using publicly available genomes becomes less useful with increasing phylogenetic distance.

Feasibility of non-NGS detection methods is another important consideration when identifying candidate wild hosts for virus ecology studies, as wild plant tissues typically contain more inhibitors of RT and PCR reactions (Lacroix et al., 2016) which could make subsequent work challenging and halt progress at the virome characterization stage. At the same time, costs must be kept low, which is equally challenging when dealing with frequent co-infections and the need to detect multiple viruses from the same sample. For our candidate hosts, we explored the use of general and virus-specific primers in RT reactions, and looked for evidence of inhibitors by diluting RNA as previously recommended (Lacroix et al., 2016). For CABYV, use of random hexamers in the RT reaction resulted in detection of 12/17 of the Illumina detections, and additionally detected five possible infections that were missed by Illumina sequencing (Figure 5B). The same pattern was seen for ToCV detections (Figure 5B). Use of previously published, virus-specific primers on undiluted or 1/10 diluted RNA [to reduce the possible influence of inhibitors (Lacroix et al., 2016)] only slightly improved detection efficiency of RT-PCR vs. Illumina for CABYV, and only for the total RNA extractions. These data indicate that for detection of multiple viruses identified in our system, use of random hexamers is an acceptable, lower-cost alternative to virus-specific RT reactions. Additionally, our target hosts are not overly problematic with regard to inhibitors. Use of a RT reagent specifically labeled for use with inhibitor-prone samples was sufficient for robust detection.

## Conclusion

Viruses are ubiquitous microbial associates within all habitats that support cellular life and represent the most important emerging infectious diseases of plants. By quantifying pathosystem characteristics across a representative sample of plant virus ecology literature, we identified key knowledge gaps that hinder our understanding of the diversity, prevalence, and ecological roles of plant viruses outside of agricultural systems. In particular, we found major need to increase the number of studies focusing on the undomesticated, dicot perennial plants that form the basis of most native communities. Our case study directly addresses this need by evaluating the tractability of three keystone dicot perennials in fragmented semi-arid plant communities in southern California (United States), which is a Mediterranean-climate biodiversity hotspot (Myers et al., 2000).

Mediterranean-climate regions like this experience a unique off-set of temperature and precipitation peaks (winter rains, summer drought) that offers advantages for agriculture (e.g., ripening fruit experience less fungal disease) and human populations. As a result, these regions provide dramatic and clear examples of interfaces between annual crops and diverse native perennial vegetation, as likewise seen in South Africa and France (Bernardo et al., 2018) and Australia (Vincent et al., 2014), as well as with urbanized areas. Here, we expand the suite of possible dicot perennial study systems while providing a first glimpse into the ecology of plant viruses in North American Mediterranean-climate region communities. In doing so, we found evidence that some crop-associated viruses (e.g., CABYV) are strongly associated with our candidate hosts, while others (PRSV and ZYMV) were detected only sporadically. Our detection of ToCV demonstrates the usefulness of perennials as subjects for virus monitoring. And multiple instances of unusual virus–plant associations (based on host range studies with crops) indicate that our three candidate hosts may be strongly connected via their shared insect vector communities. Additionally, our study lays the groundwork for controlled field and greenhouse studies to explore virus effects on wild plant performance under variable environmental conditions – all of which are logistically feasible because our hosts were selected based on the criteria defined in Figure 4. Perennials can suffer multi-year fitness impacts of viruses, especially viruses whose prevalence in wild communities is driven by amplification in adjacent annual cropping systems (Alexander et al., 2013; Malmstrom and Alexander, 2016; Malmstrom et al., 2017). Alternatively, known and novel viruses may contribute to the drought and heat tolerance characteristics of their hosts (Xu et al., 2008; Davis et al., 2015; Carr, 2017). Both possibilities remain underexplored for wild plants, but manipulative studies with our candidate hosts and their pathogens are now possible with the resources provided by our case study.

## Author Contributions

TMS and KEM secured funding from the Shipley-Skinner Endowment Fund for this research, developed field sampling methods, collected plant and insect materials, and assisted with dsRNA extractions. TMS and KEM also assembled the literature databases and performed the quantitative synthesis. PS adapted molecular methods from published literature, performed dsRNA extraction, library preparation, and Illumina data analysis. CD assisted PS and TMS with alignments. CMM helped to develop molecular methods, assisted with bioinformatics workflow improvement, performed model selections for phylogenetic analyses, generated radial trees, and performed the network analysis. All authors discussed and interpreted the data and results, and collectively wrote the manuscript.

## Conflict of Interest Statement

The authors declare that the research was conducted in the absence of any commercial or financial relationships that could be construed as a potential conflict of interest.

## Footnotes

1 https://www.ncbi.nlm.nih.gov/genomes/GenomesGroup.cgi?taxid=10239&host=plants
