# Supplementary material for: Addressing Research Needs in the Field of Plant Virus Ecology by Defining Knowledge Gaps and Developing Wild Dicot Study Systems
Source: Front Microbiol. 2019 Jan 9;9:3305. doi: 10.3389/fmicb.2018.03305 (PMC6333650; doi:10.3389/fmicb.2018.03305)
Supplement: Supplementary file 2 [file Table_2.DOCX]

**Supplementary Data Sheet 2:** **Raw reads statistics and** **Galaxy-based workflow for analysis of Illumina reads [1]**

**Raw reads statistics**

| **Lane** | **PF Clusters** | **% of the lane** | **% Perfect barcode** | **% One mismatch barcode** | **Yield (Mbases)** | **% PF Clusters** | **% >= Q30 bases** | **Mean Quality Score** |
| --- | --- | --- | --- | --- | --- | --- | --- | --- |
| Batch1 | 49,553,670 | 100.00 | 98.60 | 1.40 | 7,532 | 86.39 | 91.52 | 33.83 |
| Batch2 | 37,444,482 | 100.00 | 98.51 | 1.49 | 5,692 | 90.09 | 92.19 | 33.98 |

**Galaxy-based workflow for analysis of Illumina reads [1]**

Note: The parameters in the whole workflow were set to default unless specifically mentioned in each step.

**Pathway for proceeding with host filtering**

Main Server (https://usegalaxy.org/)

1. Upload raw sequence data (fasq.gz files) on to galaxy using Get data-upload file-regular choose local file-start.
2. Download plant virus database in fasta format from NCBI (<https://www.ncbi.nlm.nih.gov/genomes/GenomesGroup.cgi?taxid=10239&host=plants>). Upload plant virus database.fasta into Galaxy. We chose this plant virome database, since it has the complete virome sequence for viruses that have plant hosts or those having both plant and fungus hosts.
3. Fastq groomer on all uploaded data (pair1 and pair2 for each index) from step 1. **(Input FASTQ quality scores type: Sanger & Illumina 1.8+)**

Step 4-6 are to remove sequences that can be mapped to a host genome. Since there were no actual host genomes available, we chose a close relative of the actual host whose genome has been sequenced. For a subset of samples, we also completed this workflow without mapping to host genomes.

1. Upload Cucurbita maxima genome (Cmaxima_v1.1.chr.fa.gz) from <ftp://cucurbitgenomics.org/pub/cucurbit/genome/Cucurbita_maxima/v1.1/>
2. Upload Solanum lycopersicum genome (S_lycopersicum_chromosomes.3.00.fa) from <ftp://ftp.solgenomics.net/genomes/Solanum_lycopersicum/assembly/build_3.00/S_lycopersicum_chromosomes.3.00.fa>
3. Mapping: HISAT2 on Fastq groomed data from step 3 (pair1 and pair2) against either Cucurbita maxima genome (for CF and CP samples) or Solanum lycopersicum genome (DW samples) or both genomes (for pepper samples), **write unaligned reads L and R to separate files. Parameters (spliced alignment parameters-specify spliced alignment parameters-maximum intron length 3000).** This maximum intron length is selected based on a plant mRNA seq approach and should not affect virus mapping as virus has no intron.
4. Calculating mapping statistics: Flagstat on the BAM file generated by HISAT2 in step 6 to report mapping statistics. The output includes numbers of total reads and mapped reads.
5. De novo assembly using Trinity: Trinity assembly on unaligned reads L and R from step 6. All trinity assembly parameters were kept as default in galaxy. Strand-specific library type-Not set, Minimum contig length-200bp.
6. Use “NCBI BLAST+ makeblastdb” to make a blast database for the plant virus database uploaded from step 2. Select “nucleotide” for Molecule type of input. Select the input fasta files (plant virus database.fasta), create a title for the Blast database.
7. In “NCBI BLAST+ blastn”, select the trinity assembled data from step 8 for nucleotide query sequence, select Blast database from your history-nucleotide blast database, type of blast-BlastN, output format-extended 25 columns (default).
8. Download the tab files from step 10, open it with a notepad, and select all and copy into excel.

**Pathway for proceeding without host filtering**

Main Server (https://usegalaxy.org/)

1. Upload raw sequence data (fasq.gz files) on to galaxy using Get data-upload file-regular choose local file-start.
2. Download plant virus database in fasta format from NCBI (<https://www.ncbi.nlm.nih.gov/genomes/GenomesGroup.cgi?taxid=10239&host=plants>). Upload plant virus database.fasta into Galaxy. We chose this plant virome database, since it has the complete virome sequence for viruses that have plant hosts or those having both plant and fungus hosts.
3. Fastq groomer on all uploaded data (pair1 and pair2 for each index) from step 1. **(Input FASTQ quality scores type: Sanger & Illumina 1.8+)**
4. De novo assembly using Trinity: Trinity assembly on Fastq groomed data (pair1 and pair 2 for each index) from step 3. All trinity assembly parameters were kept as default in galaxy. Strand-specific library type-Not set, Minimum contig length-200bp.
5. Use “NCBI BLAST+ makeblastdb” to make a blast database for the plant virus database uploaded from step 2. Select “nucleotide” for Molecule type of input. Select the input fasta files (plant virus database.fasta), create a title for the Blast database.
6. In “NCBI BLAST+ blastn”, select the trinity assembled data from step 4 for nucleotide query sequence, select Blast database from your history-nucleotide blast database, type of blast-BlastN, output format-extended 25 columns (default).
7. Download the tab files from step 6, open it with a notepad, and select all and copy into excel.

References:

[1] Enis Afgan, Dannon Baker, Marius van den Beek, Daniel Blankenberg, Dave Bouvier, Martin Čech, et al. (2016) The Galaxy platform for accessible, reproducible and collaborative biomedical analyses: 2016 update. Nucleic Acids Research (2016) 44(W1): W3-W10
