# Supplementary material for: Addressing Research Needs in the Field of Plant Virus Ecology by Defining Knowledge Gaps and Developing Wild Dicot Study Systems
Source: Front Microbiol. 2019 Jan 9;9:3305. doi: 10.3389/fmicb.2018.03305 (PMC6333650; doi:10.3389/fmicb.2018.03305)
Supplement: Supplementary file 3 [file Table_3.docx]

**Supplementary Data Sheet 3: Sequences used in phylogenetic analyses**

**CABYV Coat Protein:**

>MRCF3C_California_USA

ATGAATACGGTCGCGGCTAGAAATCAAAATGCAGGGAGGCGAAGGCGAAGAAATCAGCGCCCTGCGCGGCGCGACCGCGTGGTTGTGGTCAACCCCATTGGGGGACCACCGCGCGGAAGACGACAACGAAGAAACCGTCGACGCCCTAACCGAGGAGGCAGAGCTAGAAGAGGAAGCCCGGGCGAAACATTTGTATTTTCAAAGGACAATCTCACGGGCAGTTCCTCAGGAAGTATCATCTTCGGGCCGTCTCTTTCAGAGAGCCCAGCATTCAGCTCTGGAATACTTAAGGCCTACCATGAATATAAGATCATCATGGTCCAGCTGGAGTTCATCTCCGAGGCCTCTTCCACCTCCTCGGGTTCCATCTCTTATGAGTTGGACCCCCACTGCAAGCTTAGCTCCCTCCAATCCACGATTAATAAATTTGGAATCACCAAGAATGGATTGCGACGTTGGACCGCTAAGCAGATCAACGGGATGGAATGGCACGATGCAACCAAAGACCAGTTCAAGATCCTCTACAAAGGGAATGGATCTTCCTCGGTTGCGGGCAGCTTCAGAATCACCATCAAGTGCCAGGTCCAGAACCCGAAATAG

>SSCF2A_California_USA

ATGAATACGGTCGCGGCTAGAAATCAAAATGCAGGGAGGCGAAGGCGAAGAAATCAGCGCCCTGCGCGGCGCGACCGCGTGGTTGTGGTCAACCCCATTGGGGGACCACCGCGCGGAAGACGACAACGAAGAAACCGCCGACGCCCTAATCGAGGAGGCAGAGCTAGAAGAGGAAGCCCGGGCGAGACATTTGTATTTTCAAAGGACAATCTCACGGGCAGTTCCACAGGAAGTATCACCTTCGGGCCGTCTCTTTCAGAGAGCCCAGCATTCAGCTCTGGAATACTCAAGGCCTACCATGAATATAAGATCATCATGGTCCAGCTGGAGTTCATCTCCGAGGCCTCTTCCACCTCCTCGGGTTCCATCTCTTATGAGTTGGACCCCCACTGCAAGCTTAGCTCCCTCCAATCCACGATTAATAAATTTGGAATCACCAAGAATGGATTGCGACGTTGGACCGCTAAGCAGATCAACGGGATGGAATGGCACGATGCAACCAAAGACCAGTTCAAGATCCTCTACAAAGGGAATGGATCTTCCTCGGTTGCAGGCAGCTTCAGAATCACCATCAAGTGCCAGGTCCAGAACCCGAAATAG

>MRCP1_California_USA

ATGAATACGGCCGCGGCTAGAAATCAAAATGCAGGGAGGCGGAGGCGAAGAAATCAGCGCCCTACACGGCGCGACCGCGTGGTTGTGGTCAACCCCTCTGGGGGACCACCACGCGGAAGACGACAACGAAGAAACCGCCGACGCCCTAATCGAGGAGGCAGAGCTAGAGGAAGGAGCCCAGGCGAAACATTCGTATTTTCAAAGGACAATCTCACGGGCAGTTCCTCAGGAAGTATCACTTTCGGGCCATCTCTATCAGAGAGCCCAGCATTCAGCTCTGGAATACTCAAGGCCTACCATGAATATAAGATCATCATGGTCCAGCTGGAGTTCATCTCCGAGGCCTCTTCCACCTCCTCGGGTTCCATCTCTTATGAGTTGGACCCCCACTGCAAGCTTAGCTCCCTCCAATCCACGATTAATAAATTTGGAATCACCAAGAACGGATTGCGACGTTGGACAGCTAGGCAGATCAACGGGATGGAATGGCATGACGCAACTGAGGACCAATTCAAGATCCTCTACAAAGGGAACGGATCCACCTCGGTTGCGGGCAGCTTCAGGATCACCATCAAGTGCCAGGTCCAAAACCCGAAATAG

>MRCP6_California_USA

ATGAATACGGTCGCGGCTAGAAATCAAAATGCAGGGAGGCGAAGGCGAAGAAATCAGCGCCCTGCGCGGCGCGACCGCGTGGTTGTGGTCAACCCCATTGGGGGACCACCGCGCGGAAGACGACAACGAAGAAACCGCCGACGCCCTAATCGAGGAGGCAGAGCTAGAAGAGGAAGCCCGGGCGAGACATTTGTATTTTCAAAGGACAATCTCACGGGCAGTTCCACAGGAAGTATCACCTTCGGGCCGTCTCTTTCAGAGAGCCCAGCATTCAGCTCTGGAATACTCAAGGCCTACCATGAATATAAGATCATCATGGTCCAGCTGGAGTTCATCTCCGAGGCCTCTTCCACCTCCTCGGGTTCCATCTCTTATGAGTTGGACCCCCACTGCAAGCTTAGCTCCCTCCAATCCACGATTAATAAATTTGGAATCACCAAGAGTGGATTGCGACGTTGGACTGCTAAGCAGATCAACGGGATGGAATGGCACGATGCAACCGAAGACCAGTTCAAGATCCTCTACAAAGGGAATGGATCTTCCTCGGTTGCGGGCAGCTTCAGAATCACCATCAAGTGCCAGGTCCAGAACCCGAAATAG

>MRDW2_California_USA

ATGAATACGGCCGCGGCTAGAAATCAAAATGCAGGGAGGCGGAGGCGAAGAAATCAGCGCCCTACACGGCGCGACCGCGTGGTTGTGGTCAACCCCTCTGGGGGACCACCACGCGGAAGACGACAACGAAGAAACCGCCGACGCCCTAATCGAGGAGGCAGAGCTAGAGGAAGGAGCCCAGGCGAAACATTCGTATTTTCAAAGGACAATCTCACGGGCAGTTCCTCAGGAAGTATCACTTTCGGGCCATCTCTATCAGAGAGCCCAGCATTCAGCTCTGGAATACTCAAGGCCTACCATGAATATAAGATCATCATGGTCCAGCTGGAGTTCATCTCCGAGGCCTCTTCCACCTCCTCGGGTTCCATCTCTTATGAGTTGGACCCCCACTGCAAGCTTAGCTCCCTCCAATCCACGATTAATAAATTTGGAATCACCAAGAACGGATTGCGACGTTGGACAGCTAGGCAGATCAACGGGATGGAATGGCATGACGCAACTGAGGACCAATTCAAGATCCTCTACAAAGGGAACGGATCCACCTCGGTTGCGGGCAGCTTCAGGATCACCATCAAGTGCCAGGTCCAAAACCCGAAATAG

**PRSV Coat Protein:**

>MRCF3A_California_USA

TCCAAAAATGAAGCTGTGGATGCTGGTTTGAATGAAAAGCTCAATGAAAAAGAAAAACAGAAAGAAAAAGAAAAAGAAAAACAAAAAGAGAAAGAAAAAGACGATGCTAGTGACGGAAATGATGTGTCAACTAGCACAAAGACTGGAGAGAGAGATAGAGATGTCAATGTTGGGACCAGTGGAACTTTCACTGTTCCGAGAATTAAATCATTTACTGACAAGATGATTCTACCGAGAATTAAGGGAAAGACTGTCCTTAATTTAAATCATCTTCTTCAGTATAATCCGCAACAAATTGACATTTCCAACACTCGTGCCACTCAGTCACAATTTGAAAAGTGGTATGAGGGAGTGAGAAATGATTATGGCCTTAACGATAATGAAATGCAAGTGATGCTAAATGGCTTGATGGTCTGGTGTATCGAGAATGGTACGTCTCCAGACATATCTGGTGTCTGGGTCATGATGGATGGGGAAACCCAAGTTGATTATCCAATCAAGCCTTTAATTGAGCATGCTACTCCGTCATTTAGGCAAATTATGGCTCACTTTAGTAACGCGGCAGAAGCATACATTGCAAAAAGAAATGCTACTGAGAGGTACATGCCGCGATATGGAATCAAGAGGAATTTGACTGACATTAGCCTCGCTAGATACGCTTTCGATTTCTATGAGGTAAATTCAAAAACACCTGATAGGGCTCGCGAAGCTCACATGCAGATGAAAGCTGCAGCGCTGCGAAACACTAGTCGCAGAATGTTTGGTATGGACGGCAGTGTTAGTAACAGGGAAGAAAACACAGAAAGACACACAGTGGAAGATGTCAATAGAGACATGCACTCTCTCCTGGGTATGCGCAACTGA

>MRCF3B_California_USA

TCCAAAAATGAAGCTGTGGATGCTGGTTTGAATGAAAAGCTCAATGAAAAAGAAAAACAGAAAGAAAAAGAAAAAGAAAAACAAAAAGAGAAAGAAAAAGACGATGCTAGTGACGGAAATAATGTGTCAACTAGCACAAAGACTGGAGAGAGAGATAGAGATGTCAATGTTGGGACCAGTGGAACTTTCACTGTTCCGAGAATCAAATCATTTACTGACAAGATGATTCTACCGAGAATTAAGGGAAAGACTGTCCTTAATTTAAATCATCTTCTTCAGTATAATCCGCAACAAATTGACATTTCCAACACTCGTGCCACTCAGTCACAATTTGAAAAGTGGTATGAGGGAGTGAGAAATGATTATGGCCTTAACGACAATGAAATGCCAGTGATGCTAAATGGCTTGATGGTCTGGTGTATCGAGAATGGTACGTCTCCAGACATATCTGGTGTCTGGGTTATGATGGATGGGGAAACCCAAGTTGATTATCCAATCAAGCCTTTAATTGAGCATGCTACTCCGTCATTTAGGCAAATTATGGCTCACTTTAGTAACGCGGCAGAAGCATACATTGCAAAAAGAAATGCTACTGAGAGGTACATGCCGCGATATGGAATCAAGAGGAATTTGACTGACATTAGCCTCGCTAGATACGCTTTCGATTTCTATGAGGTAAATTCAAAAACACCTGATAGGGCTCGCGAAGCTCACATGCAGATGAAAGCTGCAGCGCTGCGAAACACTAGTCGCAGAATGTTTGGTATGGACGGCAGTGTTAGTAACAGGGAAGAAAACACGGAGAGACACACAGTGGAAGATGTCAATAGAGACATGCACTCTCTCCTGGGTATGCGCAACTGA

>MRCF3C_California_USA

TCTAAAAATGAAGCTGTGGATGCTGGTTTGAATGAAAAGCTCATTGAAAAAGAAAAACAGAAAGAAAAAGAAAAAGAAAAACAAAAAGAGAAAGAAAAAGATGGTGCTAGTGACGGAAATAATGTGTCAACTAGCACAAAGACTGGAGAGAGAGATAGAGATGTCAATGTTGGGACCAGTGGAACTTTCACTGTTCCGAGAATCAAATCATTTACTGACAAGATGATTCTACCGAGAATTAAGGGAAAGACTGTCCTTAATTTAAATCATCTTCTTCAGTATAATCCGCAACAAATTGACATTTCCAACACTCGTGCCACTCAGTCACAATTTGAAAAGTGGTATGAGGGAGTGAGAAATGATTATGGCCTTAACGATAATGAAATGCAAGTGATGCTAAATGGCTTGATGGTCTGGTGTATCGAGAATGGTACATCTCCAGACATATCTGGTGTCTGGGTTATGATGGATGGGGAAACCCAAGTTGATTATCCAATCAAGCCTTTAATTGAGCATGCTACTCCGTCATTTAGGCAAATTATGGCTCACTTTAGTAACGCGGCAGAAGCATACATTGCAAAAAGAAATGCTACTGAGAGGTACATGCCGCGATATGGAATCAAGAGGAATTTGACTGACATTAGCCTCGCTAGATACGCTTTCGATTTCTATGAGGTAAATTCAAAAACACCTGATAGGGCTCGCGAAGCTCACATGCAGATGAAAGCTGCAGCGCTGCGAAACACTAGTCGCAGAATGTTTGGTATGGACGGCAGTGTTAGTAACAGGGAAGAAAACACGGAGAGACACACAGTGGAAGATGTCAATAGAGACATGCACTCTCTCCTGGGTATGCGTAACTGA

>MRCP6_California_USA

CAAGATGATTCTACCGAGAATTAAGGGAAAGACTGTCCTTAATTTAAATCATCTTCTTCAGTATAATCCGCAACAAATTGACATTTCCAACACTCGTGCCACTCAGTCACAATTTGAAAAGTGGTATGAGGGAGTGAGAAATGATTATGGCCTTAACGATAATGAAATGCAAGTGATGCTAAATGGCTTGATGGTCTGGTGTATCGAGAATGGTACATCTCCAGACATATCTGGTGTCTGGGTTATGATGGATGGGGAAACCCAAGTTGATTATCCAATCAAGCCTTTAATTGAGCATGCTACTCCGTCATTTAGGCAAATTATGGCTCACTTTAGTAACGCGGCAGAAGCATACATTGCAAAAAGAAATGCTACTGAGAGGTACATGCCGCGATATGGAATCAAGAGGAATTTGACTGACATTAGCCTCGCTAGATACGCTTTCGATTTCTATGAGGTAAATTCAAAAACACCTGATAGGGCTCGCGAAGCTCACATGCAGATGAAAGCTGCAGCGCTGCGAAACACTAGTCGCAGAATGTTTGGTATGGACGGCAGTGTTAGTAACAGGGAAGAAAACACGGAGAGACACACAGTGGAAGATGTCAATAGAGACATGCACTCTCTCCTGGGTATGCGTAACTGA

**ZYMV Coat Protein:**

>SSCF2A_California_USA

TCAGGCACTCAGCCAACTGTGGCAGATGCTGGGGTTACAAGGAAAGACAAAGAAGATGACAAAGGGAAAAACAAGGACGATACAGGCTCCGGCTCAGGTGAGAAAACAGTAGCAGCTGCCACGAAGGACAAGGATGTGAATGCTGGTTCTCATGGGAAAATTGTGCCGCGTCTTTCGAAGATCACAAAGAAAATGTCATTGCCACGCGTGAAAGGAAATGTTATACTTGATATTGATCATTTGCTGGAATATAAACCGGATCAAATTGAGTTATATAACACACGAGCGTCTCATCAGCAGTTCGCCTCTTGGTTCAACCAGATTAAGACGGAATATGATTTGAACGAGCAACAGATGGGAGTTGTAATGAATGGTTTCATGGTTTGGTGCATTGAAAATGGCACTTCACCCGACATTAATGGAGTGTGGGTTATGATGGACGGAAATGAGCAAGTTGAGTATCCCTTGAAACCAATAGTTGAAAATGCAAAGCCAACGCTGCGACAAATAATGCATCATTTTTCAGATGCAGCGGAGGCATATATAGAGATGAGAAATGCAGAGGCACCATACATGCCGAGGTATGGTTTGCTTCGAAACCTACGGGATAGGAGTTTAGCACGATATGCTTTCGATTTCTATGAAGTCAATTCTAAAACTCCTGAAAGAGCCCGCGAAGCTGTTGCGCAGATGAAAGCAGCAGCTCTTAGCAATGTTTCTTCAAGGTTGTTTGGCCTTGATGGAAATGTTGCCACCACTAGCGAAGACACTGAACGGCACACTGCACGTGATGTTAATAGAAACATGCACACCTTACTAGGTGTGAATACAATGCAG

**ToCV Coat Protein:**

>SSCF1A_California_USA

CATCCTCTGGTTAGACCGTTAGATGATGGCGTAGATGACGAGGTGCAGAACTTGGGCAGGAGGGACGATTCGACATCTCTCATTCCGGCTAATCCTAATCGGTCTTCCAGTTGGGCTTTGTTGAACCCGGATACTATTAATTATAACGAGTTAAGGAAATTGAAGGTACACTCCACTAGGGGTGATACTCTTACCTTGACTCAGGAAGAGGAGTTCGAGAAGATACTCGAATCCTTTTGCAGGCGAATAATCGGTGAGACCCCGATGACGGATAAGATTTTCGCTGGTTTCTACATGTCTATGTGTCAGGCCATTGTAAACCAAGGGACCTCAGTTAAGGCAGCCGGTAATAACAGTCTTGAAAACTACTTTGAGGTAGATGGTGCGAGATTTAtAGTGGAAAACTCCGGATTTGATAAATGAGGTTAGACCCAAAATGTCCGATGTTCCAAACGCTATACGTCGGTACGCCAGAAGTCATGAAAAGATTATTCAGGACTTTATCAACTCCGGTCTTATTAAGCCTGATTATCATTTACAATTCAAACATGGCGTATTACCAAGCCATGTGTTTGGTACCGGCGATTATATAAATGGTTCGTTGATGAATATCTCAGATGATCAACTTATCTCGAACCTGCTTATGAAAAGAAACGCTTTGTGCAAGGGTAACGAGGGCAAGGAACTGTACAACGTTAACCAACTT

>SSDW4_California_USA

TGGCGCATCCTCTGGTTAGACCGTTAGATGATGGCGTACATGACGAGGTGCACAACTTGGGCAGGAGGGACGATTCGACATCTCTCATTCCGGCTAATCCTAATCGGTCTTCCAGTTGGGCTTTGTTGAACCCGGATACTATTAATTATAACGAGTTAAGGAAATTGAAGGTACACTCCACTAGGGGTGATACTCTTACCTTGACTCAGGAAGAGGAGTTCGAGAAGATACTCGAATCCTTTTGCAGGCGAATAATCGGTGAGACCCCGATGACGGATAAGATTTTCGCTGGTTTCTACATGTCTATGTGTCAGGCCATTGTAAACCAAGGGACCTCAGTTAAGGCAGCCGGTAATAACAGTCTTGAAAACTACTTTGAGGTAGATGGTGCGAGATTTAAGTGGAAAACTCCGGATTTGATAAATGAGGTTAGACCCAAAATGTCCGATGTTCCAAACGCTATACGTCGGTACGCCAGAAGTCATGAAAAGATTATTCAGGACTTTATCAACTCCGGTCTTATTAAGCCTGATTATCATTTACAATTCAAACATGGCGTATTACCAAGCCATGTGTTTGGTACCGGCGATTATATAAATGGTTCGTTGATGAATATCTCAGATGATCAACTTATCTCGAACCTGCTTATGAAAAGAAACGCTTTGTGCAAGGGTAACGAGGGCAAGGAACTGTACAACGTTAACCAACTT

>SSDW2A_California_USA

ATCCTCTGGTTAGACCGTTAGATGATGGCGTAGATGACGAGGTGCAGAACTTGGGCAGGAGGGACGATTCGACATCTCTCATTCCGGCTAATCCTAATCGGTCTTCCAGTTGGGCTTTGTTGAACCCGGATACTATTAATTATAACGAGTTAAGGAAATTGAAGGTACACTCCACTAGGGGTGATACTCTTACCTTGACTCAGGAAGAGGAGTTCGAGAAGATACTCGAATCCTTTTGCAGGCGAATAATCGGTGAGACCCCGATGACGGATAAGATTTTCGCTGGTTTCTACATGTCTATGTGTCAGGCCATTGTAAACCAAGGGACCTCAGTTAAGGCAGCCGGTAATAACAGTCTTGAAAACTACTTTGAGGTAGATGGTGCGAGATTTAAGTGGAAAACTCCGGATTTGATAAATGAGGTTAGACCCAAAATGTCCGATGTTCCAAACGCTATACGTCGGTACGCCAGAAGTCATGAAAAGATTATTCAGGACTTTATCAACTCCGGTCTTATTAAGCCTGATTATCATTTACAATTCAAACATGGCGTATTACCAAGCCATGTGTTTGGTACCGGCGATTATATAAATGGTTCGTTGATGAATATCTCAGATGATCAACTTATCTCGAACCTGCTTATGAAAAGAAACGCTTTGTGCAAGGGTAACGAGGGCAAGGAACTGTACAACGTTAACCAACTT

>SSDW2B_California_USA

TGGCGCATCCTCTGGTTAGACCGTTAGATGATGGCGTAGATGACGAGGTGCAGAACTTGGGCAGGAGGGACGATTCGACATCTCTCATTCCGGCTAATCCTAATCGGTCTTCCAGTTGGGCTTTGTTGAACCCGGATACTATTAATTATAACGAGTTAAGGAAATTGAAGGTACACTCCACTAGGGGTGATACTCTTACCTTGACTCAGGAAGAGGAGTTCGAGAAGATACTCGAATCCTTTTGCAGGCGAATAATCGGTGAGACCCCGATGACGGATAAGATTTTCGCTGGTTTCTACATGTCTATGTGTCAGGCCATTGTAAACCAAGGGACCTCAGTTAAGGCAGCCGGTAATAACAGTCTTGAAAACTACTTTGAGGTAGATGGTGCGAGATTTAAGTGGAAAACTCCGGATTTGATAAATGAGGTTAGACCCAAAATGTCCGATGTTCCAAACGCTATACGTCGGTACGCCAGAAGTCATGAAAAGATTATTCAGGACTTTATCAACTCCGGTCTTATTAAGCCTGATTATCATTTACAATTCAAACATGGCGTATTACCAAGCCATGTGTTTGGTACCGGCGATTATATAAATGGTTCGTTGATGAATATCTCAGATGATCAACTTATCTCGAACCTGCTTATGAAAAGAAACGCTTTGTGCAAGGGTAACGAGGGCAAGGAACTGTACAACGTTAACCAACTT

**ToCV Minor Coat Protein (CPm):**

>SSDW2A_California_USA

ATGGATGAAAATGAAATCTATGAGGATCAAGAGGATCTCTCTGCTCGTGGCGGTGGGGGTTTCTATTACCAGACTGTGACTTTGGGTTCCGGTGATGTGTTTCCCGTTGATTTAGCCCTAACGAGATCGGCTGAATTTGATTCGACAATTTTCTCCTTATATATTAGGTTCGTAATTAAGGAGGGGAATGTGCGTTTAAAGATCGATTTTGGAAATAATTGGGATGTGACTATGCAACAGGTGAGACTTTCTGGATGGTTTGCGGCGTTTGGTAAGATTGAAAAACCGAGAACCGCCAGGTCTGGATGGTCATATCCAATAAAATTGTTTAAAGAGGCTGGAGAAGTCATAGTGTCCATTAGTGGTTGGAGGTGTTATAAAATTTATAATGGGTATCCCGTAGATCGCGTTGATTTGGTTCTAGCAGTACCCGTTCGTGAAGTAACAGCTGATTTAAAACGACCATTGGTTGGGGATTACGTCAACTTTCATGATGTATTTACTCTTATAAAAAGTAAAAATTCTGACATCACTTTACCTAACCCGAGTCTGATATTCTACGATTCAACAAGTAAGGTTAATTTAGATGTGTCTCCAGGTGCGCGAAAACAAATTGCTCAAGTCAAGGCTGAGAAAGATCTGAACATTAAGAATCCTGAAGACTCAAAGCCTGACGTTCCTAATGATTCGCTGAGTGAAGTCGAATATCATAATCACTCTGATGTTTCCAGTGTTTTCAGATTGTATTACACATGGAGGGTTGAAAGAGATTTTGAGAGATCAGTTGAGTCGAGAATTTTCTTTCCGAATATATTTCCGACCGATTTCACAATACTTCAACAAATGTGGTATGGGACGACTGCCGGTAACGTTGAGACTTTTGTGGAGATAGGAAAAAATGAAAGGAAGTTCAACGTTGGGGTCGCCGCTTGGAAGGACAATGCATTTGGACATTTCAAATTAGATGGCCCGACTTTAGCGAAGATCTCAACAATTCCAGGCAGGTTCGTGGACCATAAAATTGAAAAAGACTCTAAAGGACATTTGATTGTGAGTATTGATAATACTGTTCTTGTGCGTACTAACAAACTGATAGTCAAACCGAGTATTCAGATCGGTTGGGAATTTCATTTACCATGGGACGCGATTGGAAAGTATGGAGTTGGTAATTTGACCAGGTTCACAGACATCATCAAACCCAATTACATCAAGTATGATGGTTCTGAAGTTCCTTTGGTGCAAACTAATACCATAGAAAGTGATCGTTCCAAGTCTGGTCATAAACTATCTCTAGTCAACCTGAAAAGTTTCAGACGCATTAGTTCTACTGCAGATTTCTTCTTCGAACCACCACCACCCTCTGAGTCCGATGACAAAACTTGGGAAGATAAGATCCAAACCGAAGTGGATATAAAGAAAGAAGAGACTATTCCGACTAATGAGGGTACCTCTTCCTCTGACTTGCCGAGTGAGAAGTCACAATTTGTTGCAGCTAATCATTATCTCTTGTCGATAGCTGAAGACAGGAACATTTTCAAAGCGGCTGTAGATCGGTACACTGGATTGGGTTTCTCAAAGGATCAAGCTGTGTTGATAATATATCAATTGGGGGTAACATTCGGCACTTCCAGAAATTGTTGCAGTGATAATTCATCGTTTCTAGTCTGGAAGACTGATACTGGAGCGCAGGTTATAATCAGAAAGGGCGCCCACTCCAGGTTTCTCAATTCACTGGTTAAATATCCTTGCAACGTGGAGAGATTGATACTACGAAGACGTAGTGCGGAGATATTGGCGTTGTTGAGGAACAAGAAATTGGCTTACCCAGACAGATTGGCAAAAAAGAAAGGGGTAAGTCAGGGATTCACATATATGGCATGTGATTTTCTCGATTACACTGCGGTAACGTTAACTCAAGAAGAGCAGTTGACTATGAATTCTGTTGTGCAGTACGTGAGACTCCATAATAAACATCGAAGAAGCATTGTGAACACGAGTCAGCTTTTCTGA

**ToCV RdRp:**

>SSDW2A_California_USA

ACTTATCGGTTGACCATTGAAGGTTGTTATATTCCCGACACTTTTTCGAGACCTGCCTCATCTCATTTGATGGCAGTCAACGATTTTATGTCAGTGGTCAACCCGGGTTTAGCTTGGATGCAATTTTTGCACAGAACTATATTGTTTGAGTATGGTGATTTTGACATGCCACCTGTTGAGAAGATGGTCCTTGATTTTTCAAAATACAAGCCTTATGTCGCAGGGGAGTTTGTTGTCTCGAAAATTCTTGGCAAAGGTGAGAGGACGAGACCGGACAGTATGAAACAGGGGATAATCTCATTGTCACATAGAAATTTTTCTGCACCGAGAATAAATGAACGTCTGGACGTTTATAAGACTGCTGAACGTCTATGTCAGAATCTCGTTAGATCTTTCGACTTTTCGAGGTTGTATGAGAACTATGATGTGATTCTTCCTGACATGTTTAAAATTGACGATTGGTTGCAGGATAGAGATGGTTCTAAGTTTGGTCGGATAAAAAGGGATATGGACCACAAATTGTTGGTCGAACAGTTTGAGAGCTTAAAATTCATGATCAAAGGGGAGATGAAACCGAAGATGGATACGTCGTCCTATACAGCTTATAATCCACCGGCGAATATCATCTATTATAACCATCTGGTAAGTATGTATTATTCCCCGTTGTTTCTGGAGGTCTTTGATAGGATATCATACTGTCTTAGTAAGAAGATAGTTATGTATTCCGGGATGAATCTAGAAACTCTCGGCACCCTGATTGGTTCTAAACTGCAGAAGCCATTGACATCATATCACACTTTGGAGATTGATTTCTCAAAGTTTGATAAGTCTCAAGGTATCCTATTTAAAGTTTATGAGGGGATGATTTACCGGTTTTTCAAGTTTTCCGAGGATTACTATACCAACATAGAGGCCACTGAATACTTCATAAAGTATCGTGGTAGGTGTGGAATCAGCGGGGAGTTGGGTGCACAAAGGAGAACGGGGTCACCGAACACTTGGTTGTCAAACACATTGGTTACTATGGGTATCATACTCAGTGTTTACGACCTGGATGATATTGATTTATTCTTAGTAAGTGGTGACGACAGTTTGATCTTTTCGAGTAAACCCTTGAGGAATAAAACTGATGAGATAAACAGAGATTTCGGTTTTGAGGCTAAGATGATAGAGAATTCAGTGCCGTATTTTTGCTCCAAATATATCATCAGTGATAGAGGAAAAATCAGAGTCGTTCCTGATCCTGTGAGGTTTTTTGAGAAGTTGTCTGTCCCAATTCGAGTTCAAGATTTTAAGAGTGACACTCTCATGCGGGAAAAATTTAGGTCTTATAAGGACTTGATGAAGGACTTTGATTACGATACAACGTGCGTTTTGGTGGATGCTTTAGTGTGTTATAGGTACAATTTACCACCGATGTGTTCATATGCAGCGTTGTGTTATATTCATTGTCTGTGTGCGAATTTTACAACTTTCAGAAGAGTCTATGAGAGCGATTTGACTGTTGTTATTTAG

**PV1 RdRp:**

>MRCF3B_TRINITY_DN1242_c1_g1_i2

CTAGCCGTAAGCGTAGAAGATTGAACGCCAGAAAAGACTACGTTTAGTAGTCCATCCGCGTGGAGCTTTGCCTGAAAAGACGACTTCTAGCCATCTCTTCTGATCCTTTGAGAACCACCCCTCAGAAGGAACAGGGTAGCAGGTTTGGTAAAAGTCCATGAATTCACAAAAGACTTTGTCCCACATTGCACCTCCTATCCACAGGCCTACAAGCCTAGAAAACGAAACTTCAAGCGTATACACGCTTGATTCCGGATAGAGTGCGAGCTTGAACCACTCACTGGTGTCACGATGAACGCGACCGTCACGATACTTCGTACCTAAGAGTTTGAACTCGGATGGATCCTTTGTTCTCTCGCACTTCTCAGGTTTGATGATCATTCCAGTTGGAACGCAATCTAGTTTTGCAACTTCCAGATCAAACTGGTCGTTTGCTCTGAACGCACTGTCGTCGCCCAGAACCCTCAGGTTTCGGATCTCTACTTGCTGGCAGTCTGCAAGATAATCGATAAGTATGTGGTTCACTACTGAGTCGATAATCTGCGTCCACCAAGATCCGGAAGGTACACCCCGGTACTTACGGAACATACGTCCGTCCGGCATTAAGATGGGAGTGTTAATGAAATACCACACCATAGCATCCCAAACGTTTCGCCATTTCTGGGCATCTTCTTTACCTACTGGTTTTCCCTCGAAGGTAGAAAAGTTGACATTCTGCTTCACAATGTCAAAAGCCACACGAATTAGCCACGCTGGTACTTTTGTGTCAAAAGACGAAAAGTCGATACCATACAGTGTCTCCCCTTCCCTTAGTTTGCAACACCATTCGGTGTAAAGGCGTTGCGCACTTTTTCCATTTAGCATCGGTGAGTTGGGATCGTTCATAAAGTCGCGATACATCAAAGGGGCGTAGAACCCTTCGACAACTAACATCTCTGCTGGATATACCCAAACCAGGCGCGTTTTTGGCTCATCAATCTCTGACATGCCACCACGCTGTCCTGCAAGACACGGAGGGAACCGCATCCTCGAAGGGTTGAAACTGGTCTTGCCACCCTGTTTCATTCGGTGTCCTAACCATCTCGCTTCATGGTAGATCTCTTCCATACAGTCGCCTTTCTTGGCTCCCATGAAAGTGGATCCTGCAGACGTGTCACGCCTGAGGAACTGACCTACCTCATGCCAATCAAGCGGGTCACGCTTGTAAGGCAGTTTGAATGCCTACTTCGCTTTGGCGATTG

>MRDW2_TRINITY_DN1448_c1_g1_i1

CCACCCCTCGTCAGGAACAGGATAGCAGGTTTGGTAAAAGTCCATGAATTCACAGAAGACTTTGTCCCACATCGCCCCGCCAATCCACAGGCCTACAAGCCTTGTGAACGAAACTTCAAGCGTATACACGCTTGATTCCGGATAGAGTGCGAGCTTGAACCACTCACTGGTGTCACGATGAACGCGACCGTCACGATATCTCGTACCTAATAGTTTGAACTCGGATGGATCCTTTGTTCTTTCACACTTCTCAGGTTTGATTATCATTCCAGTTGGAACGCAATCTAGTTTTGCAACTTCCAGGTCAAACTGGTCGTTTGCTCTGAACGCACTGTCGTCGCCCAGAACCCTCAGGTTTTGGATCTCTACTTGCTGGCAGTCTGCCAGATAATCGATAAGTATGTGGTTCACTACTGAGTCAATCATCTGCGTCCACCAAGATCCGGAAGGTACACCCCGGTACTTACGGAACATACGTCCGTCCGGCATTAAGATGGGAGTGTTAATGAAATACCACACCATAGCATCCCAAACGTTTCGCCATTTCTGTGCGTCTTCCTTGTCAACAGGTTTCCCCTCAAAGGTCGAAAATTCGATGTTCTGCCTCAGAATATCGAACGCCACACGAATCAGCCACGCAGGTACTTTTGTGTCAAAAGACGAAAAGTCGATGCCATACAGTGTCTCCCCTTCCCTTAGTTTGCAACACCATTCGGTGTAAAGGCGTTGCGCACTTTTTCCATTTAGCATCGGTGAGTTGGGATCGTTCATAAAGTCACGATACATCAAAGGAGCGTAGAATCCTTCGACAACTAACATTTCTGCTGGGTATACCCAAACCAGGCGCGTTTTCGGCTCGTCAATCTCTGACATGCCGCCACGCTGGCCTGCAAGACACGGAGGGAACCGCATCTTCGATGGGTTGAAACTTCTTTTTCCACCCTGTTTCATTCGGTGTCCTAGCCATCTCGCTTCATGGTAGATCTCTTCCATGCAGTCACCTTTCTTGGCGCCCATGAAAGTGGATCCTGCAGACGTGTCACGCCTCAGAAATTGGCCCACTTCATGCCAGTCAAGCGGTTCACGCTTGTATGGTAACTTGAAAGCTTTCTTCGCCTTTGCGATCGAACGACGCATTGACGCTTGTTGAGATGGAGACAGGTCACGGAATGTGCTACGCTCACCGGAGAACTTGTTTAGGGCGGTGTACATGCCCAGGTTGCCCTGCGGTCTGCGAGTGTAGCCACGGATGTCCTCGTAGATGTCTCGGTTGAATAACTTTAGACTCTCTCTCACCTGAGGATCGGTGTTAGAGTTAGAGCTGTAGGTTGAGTACCCCCCATACTTAGCGATCTCACGTAAGTTGGGGTCCTCGAAGTGAAGAGGGATAACATCCTCGGAAGTGGTTGACCCTGTCTTTGCGGGACGAAGGTGGAGTGCGTCAACTAAATGAGACCCCTTTCCAAGGACGTAGTGGTTGGTTGGTTCTAGAGTGAAATCTTCCATTTCGGCTCTAGAGATGAAGGAGGATAAACCCGTTTGCACGGTAAAATCGC

>MRCP6_TRINITY_DN1246_c2_g1_i1

ATGAAACAGGATGGTAGAGCCAGTTTCAACCCATCCAGGATGCGGTTCCCTCCGTGTCTTGCAGGCCAGCGTGGCGGTATGTCAGAGATTGACGATCCGAAAACGCGCCTGGTTTGGGTATATCCAGCAGAGATGTTAGTTGTCGAAGGATTCTACGCTCCTTTGATGTATCGTGACTTTATGAACGATCCCAACTCACCTATGCTAAATGGAAAAAGTGCGCAGCGCCTTTATACCGAATGGTGCTGCAAACTAAGGGAAGGGGAGACACTGTATGGTATCGACTTTTCGTCTTTTGACACAAAAGTACCAGCGTGGCTGATTCGTGTGGCTTTTGACATTGTGAAGCAGAATGTCAATTTTTCTACCTTCGAGGGAAAACCTGTTGGTAAGGAAGACGCTCAGAAATGGCGCAACGTTTGGGATGCCATGGTGTGGTATTTCATTAACACTCCCATTTTGATGCCGGACGGACGTATGTTCCGTAAGTACCGGGGTGTACCTTCCGGATCTTGGTGGACGCAGATTATCGACTCAGTAGTGAACCACATACTTATCGATTATCTTGCAGACTGCCAGCAAGTAGAGATCCGAAACCTGAGGGTTCTGGGCGACGACAGTGCGTTCAGAGCAAACGACCAGTTTGATCTGGAAGTTGCAAAACTAGATTGCGTTCCAACTGGAATGATCATCAAACCTGAGAAGTGTGAGAGAACAAAGGATCCATCCGAGTTCAAACTTTTAGGTACGAAGTATCGTGACGGTCGTGTTCATCGCGACACCAGTGAGTGGTTCAAGCTAGCACTCTATCCCGAAGGAAGCGTGTATACGCTAGACATTTCATTCACCAGACTTGTCGGCCTGTGGATAGGCGGCGCGATGTTTGATCGAGTCTTTTGTGAATTCATGGACTATTATCAAACAAGCTATCCTGTTCCTGACGAGGGGTGGTTCTCGAAGGATCAGAAGCGATGGCTCGAAGTCGTCTTTTCAGGCAAAGCCCCCAGAGGCTGGACTACAAAACGTAGTCTATTCTGGCGATCAATCTTCT

>MRCP1_TRINITY_DN2411_c0_g1_i2

TCTCTCACCATCACAACAAGCCTCGATGCGCCGTTCTATCGGCAAAGCGAAAAAGGCATTCAAGTTGCCATACAAGCGTGAACCGCTTGATTGGCATGAGGTAGGTCAATTCCTCAGGCGTGACACGTCTGCAGGATCCACTTTCATGGGAGCCAAGAAAGGCGACTGTATGGAAGAGATCTACCATGAAGCAAGATGGTTAGGACACCGAATGAAACAGGGTGGTAAAACTAGTTTCAACCCTTCGAAGATGCGGTTCCCTCCGTGTCTTGCAGGACAGCGTGGTGGCATGTCAGAGATTGATGAGCCAAAAACGCGCCTGGTTTGGGTATATCCAGCAGAGATGTTAGTTGTCGAAGGATTCTACGCTCCTTTGATGTATCGTGACTTTATGAACGATCCCAACTCACCAATGCTAAATGGAAAAAGTGCGCAACGCCTTTACACCGAATGGTGTTGCAAACTAAGGGAAGGGGAGACACTGTATGGTATCGACTTTTCGTCTTTTGACACAAAAGTACCTGCGTGGCTGATTCGAGTGGCTTTTGACATAGTTAAGCAGAATGTCAACTTTTCTACCTTCGAGGGAAAACCTGTTGGTAAAGAAGACGCTCAGAAATGGCGCAACGTTTGGGATGCCATGGTGTGGTATTTCATTAACACTCCCATTTTGATGCCGGACGGACGTATGTTCCGTAAGTACCGGGGTGTACCTTCCGGATCTTGGTGGACGCAGATTATCGACTCAGTAGTGAACCACATACTTATCGATTATCTTGCAGACTGCCAGCAAGTAGAGATCCGAAACCTGAGGGTTCTGGGCGACGACAGTGCGTTCAGAGCAAACGACCAGTTTGATCTGGAAGTTGCAAAACTAGATTGCGTTCCAACTGGAATGATCATCAAACCTGAGAAGTGTGAGAGAACAAAGGATCCATCCGAGTTCAAACTCTTAGGTACGAAGTATCGTGACGGTCGCGTTCATCGTGACACCAGTGAGTGGTTCAAGCTCGCACTCTATCCGGAATCAAGCGTGTATACGCTTGAAGTTTCGTTCACTAGGCTTGTAGGCCTGTGGATAGGAGGTGCAATGTGGGACAAAGTCTTTTGTGAATTCATGGACTTTTACCAAACCTGCTACCCTGTTCCTTCTGAGGGGTGGTTCTCAAAGGATCAGAAGAGATGGCTAGAAGTCGTCTTTTCAGGCAAAGC

>MRCF3C_TRINITY_DN5133_c1_g1_i2

GCGATTTTACCGTGCAAACGGGTTTATCCTCCTTCATCTCTGGAGCCGAAATGGAAGATTTCACTTCAGAACCAACCAACCACTACGTCCTTGGAAAGGGGTCTCATTTAGTTGACGCACTCCACCTTCGTCCCGCAAAGACAGGGTCAACCACTTCCGAGGATGTTATCCCTCTTCACTTCGAGGACCCCAACTTACGTGAAATCGCTAAGTATGGGGGGTACTCAACCTACAGCTCTAACTCTAACACCGATCCTCAGGTGAGAGAGAGTCTAAAGTTATTCAACCGAGACGTCTACGAGGACATCCGTGGCTTCACTCGCCGCCCACAAGGTAACGTGGGTATGTACACTGCTTTAAACAAGTTCTCAGGCGAGAAAAGCACATTCAAGAGTCTCTCACCATCTCAGCAATCTTCGATGCGGCGTTCAATCGCCAAGGCAAAGAAGGCTTTCAAATTGCCTTACAAGCGTGACCCGCTTGATTGGCATGAGGTAGGTCAATTCCTCAGGCGTGACACGTCTGCAGGATCCACTTTCATGGGAGCCAAGAAAGGCGACTGTATGGAAGAGATCTACCATGAAGCGAGATGGTTAGGACACCGAATGAAACAGGGTGGCAAGACCAGTTTCAACCCTTCGAGGATGCGGTTCCCTCCGTGTCTTGCAGGACAGCGTGGTGGCATGTCAGAGATTGATGAGCCAAAAACGCGCCTGGTTTGGGTATATCCAGCAGAGATGTTAGTTGTCGAAGGATTCTACGCTCCTTTGATGTATCGTGACTTTATGAACGATCCCAACTCACCAATGCTAAATGGAAAAAGTGCGCAGCGCCTTTACACCGAATGGTGCTGTAAACTAAGGGAAGGGGAGACACTATATGGTATCGACTTTTCGTCTTTTGACACAAAAGTACCTGCGTGGCTAATTCGAGTGGCTTTTGACATAGTTAAGCAGAATGTCAACTTTTCTACCTTCGAGGGAAAACCTGTTGGTAAAGAAGACGCTCAGAAATGGCGCAACGTTTGGGATGCCATGGTGTGGTATTTCATTAACACTCCCATTTTGATGCCGGACGGACGTATGTTCCGTAAGTACCGGGGTGTACCTTCCGGATCTTGGTGGACGCAGATTATCGACTCAGTAGTGAACCACATACTTATCGATTATCTTGCAGACTGCCAGCAAGTAGAGATCCGAAACCTGAGGGTTCTGGGCGACGACAGTGCGTTCAGAGCAAACGACCAGTTTGATCTGGAAGTTGCAAAACTAGATTGCGTTCCAACTGGAATGATCATCAAACCTGAGAAGTGTGAGAGAACAAAGGATCCATCCGAGTTCAAACTTTTAGGTACGAAGTATCGTGACGGTCGCGTTCATCGTGACACCAGTGAGTGGTTCAAGCTCGCACTCTATCCGGAATCAAGCGTGTATACGCTTGAAGTTTCGTTTTCTAGGCTTGTAGGCCTGTGGATAGGAGGTGCAATGTGGGACAAAGTCTTTTGTGAATTCATGGACTTCTACCAAACCTGCTACCCTGTTCCTTCTGAGGGGTGGTTCTCAAAGGATCAGAAGAGATGGCTAGAAGTCGTCTTTTCAGGCAAAGCTCCACGCGGATGGACTACTAAACGTAGTCTTTTCTGGCGTTCAATCTTCTACGCTTACGGCTAGGAGTGA

>MRCF2C_TRINITY_DN798_c0_g1_i1

GTCACTCCTAGCCGTAAGCGTAGAAGATTGAACGCCAGAAAAGACTACGTTTAGTAGTCCATCCGCGTGGAGCTTTGCCTGAAAAGACGACTTCTAGCCATCTCTTCTGATCCTTTGAGAACCACCCCTCAGAAGGAACAGGGTAGCAGGTTTGGTAAAAGTCCATGAATTCACAAAAGACTTTGTCCCACATTGCACCTCCTATCCACAGGCCTACAAGCCTAGAAAACGAAACTTCAAGCGTATACACGCTTGATTCCGGATAGAGTGCGAGCTTGAACCACTCACTGGTGTCACGATGAACGCGACCGTCACGATACTTCGTACCTAAAAGTTTGAACTCGGATGGATCCTTTGTTCTCTCACACTTCTCAGGTTTGATGATCATTCCAGTTGGAACGCAATCTAGTTTTGCAACTTCCAGATCAAACTGGTCGTTTGCTCTGAACGCACTGTCGTCGCCCAGAACCCTCAGGTTTCGGATCTCTACTTGCTGGCAGTCTGCAAGATAATCGATAAGTATGTGGTTCACTACTGAGTCGATAATCTGCGTCCACCAAGATCCGGAAGGTACACCCCGGTACTTACGGAACATACGTCCGTCCGGCATCAAAATGGGAGTGTTAATGAAATACCACACCATGGCATCCCAAACGTTGCGCCATTTCTGAGCGTCTTCTTTACCAACAGGTTTTCCCTCGAAGGTAGAAAAGTTGACATTCTGCTTAACAATGTCAAAAGCCACACGAATCAGCCACGCTGGTACTTTTGTGTCAAAAGACGAAAAGTCGATACCATACAGTGTCTCCCCTTCCCTTAGTTTGCAACACCATTCGGTGTAAAGGCGTTGCGCACTTTTTCCATTTAGCATAGGTGAGTTGGGATCGTTCATAAAGTCGCGATACATCAAAGGGGCGTAGAACCCTTCGACAACTAACATCTCTGCTGGATATACCCAAACCAGGCGCGTTTTTGG

>MRCF2B_TRINITY_DN970_c1_g1_i1

TTTCTACCTTCGAGGGAAAACCTGTTGGTAAAGAAGATGCTCAGAAATGGCGCAACGTTTGGGATGCCATGGTGTGGTATTTCATTAACACTCCCATTTTGATGCCGGACGGACGTATGTTCCGTAAGTACCGGGGTGTACCTTCCGGATCTTGGTGGACGCAGATTATCGACTCAGTAGTGAACCACATACTTATCGATTATCTTGCAGACTGCCAGCAAGTAGAGATCCGAAACCTGAGGGTTCTGGGCGACGACAGTGCGTTCAGAGCAAACGACCAGTTTGATCTGGAAGTTGCAAAACTAGATTGCGTTCCAACTGGAATGATCATCAAACCTGAGAAGTGTGAGAGAACAAAGGATCCATCCGAGTTCAAACTTTTAGGTACGAAGTATCGTGACGGTCGCGTTCATCGTGACACCAGTGAGTGGTTCAAGCTCGCACTCTATCCGGAATCAAGCGTGTATACGCTTGAAGTTTCGTTTACTAGGCTTGTAGGCCTGTGGATAGGAGGTGCAATGTGGGACAAAGTCTTTTGTGAATTCATGGACTTCTACCAAACCTGCTACCCTGTTCCTTCTGAGGGGTGGTTCTCAAAGGATCAGAAGAGATGGCTAGAAGTCGTCTTTTCAGGCAAAGCTCCACGCGGATGGACTACCAAGCGTAGTCTTTT

>MRCF2A_TRINITY_DN1305_c0_g2_i1

CATTCAAGAGTCTTTCACCATCTCAGCAATCTTCGATGCGGCGTTCAATCGCTAAAGCGAAGAAGGCATTCAAGTTGCCATACAAGCGTGAACCGCTTGATTGGCACGAGGTGGGTCAGTTCCTCAGACGTGACACGTCTGCAGGTTCAACTTTCATGGGCGCCAAGAAGGGTGACTGCATGGAAGAGATCTACCATGAGGCAAGATGGTTAGGACACAGAATGAAACAGGATGGTAAATCTAGTTTCAACCCTTCGAAGATGCGGTTCCCTCCGTGTCTTGCAGGCCAGCGTGGCGGCATGTCAGAGATTGACGAGCCGAAAACGCGCCTGGTTTGGGTATATCCAGCAGAGATGTTAGTTGTCGAAGGATTCTACGCTCCTTTGATGTATCGTGACTTTATGAACGATCCCAACTCACCTATGCTAAATGGAAAAAGTGCGCAACGCCTTTACACCGAATGGTGTTGCAAACTAAGGGAAGGGGAGACACTATATGGCATCGACTTTTCGTCTTTTGACACAAAAGTACCTGCGTGGCTAATTCGAGTGGCTTTTGACATAGTTAAGCAGAATGTCAACTTTTCTACCTTCGAGGGAAAACCAGTAGGTAAAGAAGATGCCCAGAAATGGCGAAACGTTTGGGATGCTATGGTGTGGTATTTCATTAACACTCCCATTTTGATGCCGGACGGACGTATGTTCCGTAAGTACCGGGGTGTACCTTCCGGATCTTGGTGGACGCAGATTATCGACTCAGTAGTGAACCACATACTTATCGATTATCTTGCAGACTGCCAGCAAGTAGAGATCCGAAACCTGAGGGTTCTGGGCGACGACAGTGCGTTCAGAGCAAACGACCAGTTTGATCTGGAAGTTGCAAAACTAGATTGCGTTCCAACTGGAATGATCATCAAACCTGAGAAGTGTGAGAGAACAAAGGATCCATCCGAGTTCAAGCTCTTAGGTACGAAGTATCGTGACGGTCGCGTTCATCGTGACACCAGTGAGTGGTTCAAGCTCGCACTCTATCCGGAATCAAGCGTGTATACGCTTGAAGTTTCGTTCACTAGGCTAATAGGCCTGTGGATAGGTGGAGCAATGTGGGACAA

>MRCF1C_TRINITY_DN6587_c2_g1_i5

GCCCCGCGCTTTTACCGTGTAAACGGGTTTAACCTCCTTTTTCTCTGAAGCCGAAATGGAAGATTTCACTTCAGAACCAACCAACCACTACGTCCTTGGAAAGGGGTCTCATTTAGTTGACGCACTCCACCTTCGTCCCGCAAAGACAGGGTCAACCACTTCCGAGGATGTTATCCCTCTTCACTTCGAGGACCCCAACTTACGTGAAATCGCTAAGTATGGGGGGTACTCAACCTACAGCTCTAACTCTAACACCGATCCTCAGGTGAGAGAGAGTCTAAAGTTATTCAACCGAGACGTCTACGAGGACATCCGTGGCTTCACTCGCCGCCCACAAGGTAACGTGGGTATGTACACTGCACTAAACAAGTTTGCAGGCGAGAAAAGCTCATTCTCAAGTCTCTCTCCATCACAAAAATCTTCGATGCGGCGTTCGATCGCCAAAGCAAAGAAGGCTTTCAAGTTGCCATACAAGCGTGAACCGCTTGATTGGCACGAGGTGGGTCAGTTCCTCAGACGTGACACGTCTGCAGGTTCAACTTTCATGGGCGCCAAGAAGGGTGACTGCATGGAAGAGATCTACCATGAAGCAAGATGGTTAGGACACCGAATGAAACAGGGTGGCAAGACCAGTTTCAACCCTTCGAGGATGCGGTTCCCTCCGTGTCTTGCAGGACAGCGTGGTGGCATGTCAGAGATTGATGAGCCAAAAACGCGCCTGGTTTGGGTATATCCAGCAGAGATGTTAGTTGTCGAAGGGTTCTACGCCCCTTTGATGTATCGCGACTTTATGAACGATCCCAACTCACCGATGCTAAATGGAAAAAGTGCGCAACGCCTTTACACCGAATGGTGTTGCAAACTAAGGGAAGGGGAGACACTGTATGGTATCGACTTTTCGTCTTTTGACACAAAAGTACCTGCGTGGCTAATTCGAGTGGCTTTTGACATAGTTAAGCAGAATGTCAACTTTTCTACCTTCGAGGGAAAACCAGTAGGTAAAGAAGATGCCCAGAAATGGCGAAACGTTTGGGATGCTATGGTGTGGTATTTCATTAACACTCCCATTTTGATGCCGGACGGACGTATGTTCCGTAAGTACCGGGGTGTACCTTCCGGATCTTGGTGGACGCAGATTATCGACTCAGTAGTGAACCACATACTTATCGATTATCTTGCAGACTGCCAGCAAGTAGAGATCCGAAACCTGAGGGTTCTGGGCGACGACAGTGCGTTCAGAGCAAACGACCAGTTTGATCTGGAAGTTGCAAAACTAGATTGCGTTCCAACTGGAATGATCATCAAACCTGAGAAGTGTGAGAGAACAAAGGATCCATCCGAGTTCAAACTTTTAGGTACGAAGTATCGTGACGGTCGCGTTCATCGTGACACCAGTGAGTGGTTCAAGCTCGCACTCTATCCGGAATCAAGCGTGTATACGCTTGAAGTTTCGTTCACTAGGCTTGTAGGCCTGTGGATAGGAGGTGCAATGTGGGACAAAGTCTTTTGTGAATTCATGGACTTTTACCAAACCTGCTACCCTGTTCCTTCTGAGGGGTGGTTCTCAAAGGATCAGAAGAGATGGCTAGAAGTCGTCTTTTCAGGCAAAGCTCCACGCGGATGGACTACTAAACGTAGTCTTTTCTGGCGTTCAATCTTCTACGCTTACGGCTAGGAGTGAC

>SSDW4_TRINITY_DN1723_c0_g1_i1

TCTAAACGTTCTCGACAAAAGTGGAGAAACGTGTGGGATGGTATGGTGTGGTATTTCATTAACACTCCCATCTTAATGCCGGACGGACGTATGTTCCGTAAGTACCGGGGTGTACCTTCCGGATCTTGGTGGACGCAGATTATCGACTCAGTAGTGAACCACATACTTATCGATTATCTGGCAGACTGCCAGCAAGTAGAGATCCGAAACCTGAGGGTTCTGGGCGACGACAGTGCGTTCAGAGCAAACGACCAGTTTGATCTGGAAGTTGCAAAACTAGATTGCGTTCCAACTGGAATGATCATCAAACCTGAGAAGTGTGAGAGAACAAAGGACCCATCCGAGTTCAAACTATTAGGTACGAAGTATCGTGACGGTCGCGTTCATCGTGACACCAGTGAGTGGTTAAAGCTCGCACTCTATCCGGAATC

>SSCF3A_TRINITY_DN958_c0_g1_i1

CAGAAATGGCGAAACGTTTGGGATGCTATGGTGTGGTATTTCATTAACACTCCCATCTTAATGCCGGACGGACGTATGTTCCGTAAGTACCGGGGTGTACCTTCCGGATCTTGGTGGACGCAGATTATCGACTCAGTAGTGAACCACATACTTATCGATTATCTTGCAGACTGCCAGCAAGTAGAGATCCGAAACCTGAGGGTTCTGGGCGACGACAGTGCGTTCAGAGCAAACGACCAGTTTGATCTGGAAGTTGCAAAACTAGATTGCGTTCCAACTGGAATGATCATCAAACCTGAGAAGTGTGAGAGAACAAAGGATCCATCCGAGTTCAAACTTTTAGGTACGAAGTATCGTGACGGTCGCGTTCATCGCGACACCAGTGAGTGGTTCAAGCTCGCACTCTATCCGGAATCAAGCGTGTATACGCTTGAAGTTTCGTTTACTAGGCTTGTAGGCCTGTGGATAGGAGGTGCAATGTGGGACAAAGTCTTTTGTGAATTCATGGACTTTTACCAAACCTGCTACCCTGTTCCTTCTGAGGGGTGGTTCTCAAAGGATCAGAAGAGATGGCTAGAAGTCGTCTTTTCAGGCAAAGCTCCACGCGGATGGACTACTAAACGTAGTCTTTTCTGGCGTTCAATCTTCTACGCTTACGGCTAGGAGTGAC

>SSCF2B_TRINITY_DN1163_c1_g1_i2

AAACCTGTTGACAAAGTTGACGCACAGAAATGGCGAAACGTTTGGGATGCTATGGTGTGGTATTTCATTAACACTCCCATTTTGATGCCGGACGGACGTATGTTCCGTAAGTACCGGGGTGTACCTTCCGGATCTTGGTGGACGCAGATTATCGACTCAGTAGTGAACCACATACTTATCGATTATCTTGCAGACTGCCAGCAAGTAGAGATCCGAAACCTGAGGGTTCTGGGCGACGACAGTGCGTTCAGAGCAAACGACCAGTTTGATCTGGAAGTTGCAAAACTAGATTGCGTTCCAACTGGAATGATCATCAAACCTGAGAAGTGTGAGAGAACAAAGGACCCATCCGAGTTCAAACTCTTAGGTACGAAGTATCGTGACGGTCGCGTTCATCGTGACACCAGTGAGTGGTTCAAGCTCGCACTCTATCCGGAATCAAGCGTGTATACGCTTGAAGTTTCGTTCACTAGGCTTGTAGGCCTGTGGATAGGAGGTGCAATGTGGGACAAAGTCTTTTGTGAATTCATGGACTTTTACCAAACCTGCTACCCTGTTCCTTCTGAGGGGTGGTTCTCAAAGGATCAGAAGAGATGGCTAGAAGTCGTCTTTTCAGGCAAAGCTCCACGCGGATGGACTACTAAACGTAGTCTTTTCTGGCGTTCAATCTTCTACGCTTACGGCTAGGAGTGAC

>SSDW2B_TRINITY_DN6041_c0_g1_i1

GATACTTCTAGCGTATACACGCTTGACTCAGGATAGAGTGCGAGCTTGAACCACTCACTGGTGTCACGATACACGCGACCGTCACGATACTTTGTACCCAAAAGTTTGAAATCCGAAGGATTTGTGCTCTTGTCACACTTCTCAGGTTTGATAATCATTCCAGTTGGCTCACAGTCTATTTTTGCAGCTTCCAGATCGAACTGGTCGTTGCCTCTGAACGCACTGTCGTCGCCCAGAACCCTCAGGTTTCGGATCTCTACTTGCTGGCAGTCTGCAAGATAATCGATAAGTATGTGGTTCACTACTGAGTCGATCATCTGTGTCCACCAAGATCCGGAAGGAACACCCCGGTATTTACGGAACATACGTCCGTCAGGCATGAGGATGGGAGTGTTAATGAAATACCACACCATAGCATCCCAAACGTTTCGCCATTTCTGGGCATC

>SSCF2A_TRINITY_DN4363_c2_g4_i5

CGAGACATCTACGAGGACATCCGTGGTTTCACTCGCCGCCCACAAGGTAACGTGGGTATGTACACTGCACTAAACAAGTTTGCAGGCGAGAAAAGCTCATTCTCCAGTCTCTCTCCATCACAAAAATCTTCGATGCGGCGTTCTATCGCTAAAGCAAAGAAGGCTTTCAAGTTGCCATACAAGCGTGAACCGCTTGATTGGCATGAAGTAGGTCAATTCCTCAGGCGTGATACGTCTGCAGGGTCAACCTTCATGGGCGCCAAGAAAGGCGATTGCATGGAGGAAATCTACCACGAAGCACGATGGTTAGGACACAGAATGAAACAGGATGGTAAATCTAGTTTCAACCCTTCGAAGATGCGGTTCCCTCCGTGTCTTGCAGGACAGCGTGGTGGCATGTCAGAGATTGATGAGCCAAAAACGCGCCTGGTTTGGGTATATCCAGCAGAGATGTTAGTTGTCGAAGGGTTCTACGCCCCTTTGATGTATCGTGACTTTATGAACGATCCCAACTCACCGATGCTAAATGGAAAAAGTGCGCAGCGCCTTTACACCGAATGGTGCTGTAAACTAAGGGAAGGGGAGACACTATATGGTATCGACTTTTCGTCTTTTGACACAAAAGTACCAGCGTGGCTGATTCGTGTGGCCTTTGACATTGTGAAGCAGAATGTCAACTTTTCTACCTTCGAGGGAAAACCAGTAGGTAAAGAAGATGCCCAGAAATGGCGAAACGTTTGGGATGCTATGGTGTGGTATTTCATTAACACTCCCATTTTGATGCCGGACGGACGTATGTTCCGTAAGTACCGGGGTGTACCTTCCGGATCTTGGTGGACGCAGATCATCGACTCAGTAGTGAACCACATACTTATCGATTATCTTGCAGACTGCCAGCAAGTAGAGATCCGAAACCTGAGGGTTCTGGGCGACGACAGTGCGTTCAGAGGCAATGACCAGTTCGACCTGGAAATTGCAAAAATAGACTGTGAGCCAACTGGAATGATCATCAAACCTGAGAAGTGTGAAAGAACAAAGGATCCATCCGAGTTCAAGCTCTTAGGTACGAAGTATCGTGACGGTCGCGTTCATCGTGACACCAGTGAGTGGTTCAAGCTCGCACTCTATCCGGAATC

>SSCF1B_TRINITY_DN3908_c0_g1_i3

GCGGCGTTCTATCGCTAAAGCAAAGAAGGCTTTCAAGTTGCCATACAAGCGTGAACCGCTTGATTGGCATGAGGTGGGTCAGTTCCTCAGACGTGACACGTCTGCAGGATCCACTTTCATGGGAGCCAAGAAAGGCGACTGCATGGAGGAGATCTACCATGAAGCGAGATGGTTAGGACACCGAATGAAACAGGGTGGTAAGGCCAGTTTCAACCCTTCGAAGATGCGGTTCCCTCCGTGTCTTGCAGGACAGCGTGGTGGCATGTCGGAGATCGATGAGCCAAAAACGCGCCTGGTTTGGGTATATCCAGCAGAGATGTTAGTTGTCGAAGGATTCTACGCTCCTTTGATGTATCGCGACTTTATGAACGATCCAAACTCACCGATGCTAAATGGAAAAAGTGCGCAACGCCTCTACACCGAGTGGTGTTGCAAACTAAGGGAAGGGGAGACACTATACGGTATCGACTTTTCGTCTTTTGACACAAAGGTGCCAGCGTGGCTGATTCGTGTGGCTTTTGACATTGTGAAGCAGAATGTCAACTTTTCTACCTTCGAGGGAAAACCAGTAGGTAAAGAAGATGCCCAGAAGTGGCGAAACGTTTGGGATGCTATGGTGTGGTATTTCATTAACACTCCCATTTTGATGCCGGACGGACGTATGTTCCGTAAGTACCGGGGTGTACCTTCCGGATCTTGGTGGACGCAGATTATCGACTCAGTAGTGAACCACATACTTATCGATTATCTTGCAGACTGCCAGCAAGTAGAGATCCGAAACCTGAGGGTTCTGGGCGACGACAGTGCGTTCAGAGCAAACGACCAGTTTGATCTGGAAGTTGCAAAACTAGATTGCGTGCCAACTGGAATGATAATCAAACCTGAGAAGTGTGAGAGAACAAAGGATCCATCCGAGTTCAAACTTTTAGGTACAAAGTATCGTGACGGTCGCGTTCATCGTGACACCAGTGAGTGGTTCAAGCTCGCACTCTATCCGGAATCAAGCGTGTATACGCTTGAAGTTTCGTTCACTAGGCTTGTAGGCCTGTGGATAGGAGGTGCAATGTGGGACAAAGTCTTTTGTGAATTCATGGACTTTTACCAAACCTGCTACCCTGTTCCTTCTGAGGGGTGGTTCTCAAAGGATCAGAAGAGATGGCTTGAAGTCGTCTTTTCAGGCAAAGCTCCACGCGGATGGACTACAAAACGTAGTCTTTTCTGGCGTTCAATCTTCTACGCTTACGGCTAGGAGTGAC

>SSCF1A_TRINITY_DN2891_c0_g1_i4

TCACTCCTAGCCATAAGCGTAGAAGATTGAACGCCAGAAAAGACTACGTTTAGTAGTCCATCCGCGTGGAGCTTTGCCTGAAAAGACGACTTCTAGCCATCTCTTCTGATCCTTTGAGAACCACCCCTCAGAAGGAACAGGGTAGCAGGTTTGGTAAAAGTCCATGAATTCACAAAAGACTTTGTCCCACATTGCACCTCCTATCCACAGGCCTACAAGCCTAGTGAACGAAACTTCAAGCGTATACACGCTTGATTCCGGATAGAGTGCGAGCTTGAACCACTCACTGGTGTCACGATGAACGCGACCGTCACGATACTTCGTACCTAGAAGTTTGAACTCGGATGGATCCTTTGTTCTCTCGCACTTCTCAGGTTTGATGATCATTCCAGTTGGAACGCAATCTAGTTTTGCAACTTCCAGATCAAACTGGTCGTTTGCTCTGAACGCACTGTCGTCGCCCAGAACCCTCAGGTTTCGGATCTCTACTTGCTGGCAGTCCGCAAGATAATCGATAAGTATGTGGTTCACTACTGAGTCAATAATCTGCGTCCACCAAGATCCGGAAGGTACACCCCGGTACTTACGGAACATACGTCCGTCCGGCATCAAGATGGGAGTGTTAATGAAATACCACACCATAGCATCCCAAACGTTTCGCCACTTCTGGGCATCTTCTTTACCTACTGGTTTTCCCTCGAAGGTAGAAAAGTTGACATTCTGTTTCACAATGTCAAAAGCCACACGAATTAGCCACGCTGGTACTTTTGTGTCAAAAGACGAAAAGTCGATACCATACAGTGTCTCCCCTTCCCTTAGTTTACAGCACCACTCGGTGTAGAGGCGCTGCGCACTTTTTCCATTTAGCATCGGTGAGTTGGGATCGTTCATAAAGTCGCGATACATCAAAGGAGCGTAGAATCCTTCGACAACTAACATCTCTGCTGGATATACCCAAACCAGGCGCGTTTTTGGCTCGTCAATCTCTGACATGCCACCACGCTGTCCTGCAAGACACGGAGGGAACCGCATCCTCGAAGGGTTGAAACTGGTCTTGCCACCCTGTTTCATTCGGTGTCCTAACCATCTCGCCTCATGGTAGATCTCCTCCATACAGTCGCCTTTCTTGGCTCCCATAAAGGTGGACCCTGCAGACGTGTCACGCCTGAGGAATTGACCTACCTCATGCCAATCAAGCGGGTCACGCTTGTATGGCAGCTTGAATGCTTTCTTCGCTTTGGCGATTGAACGCCGCATCGATGATTGCTGAGATGGAGACAGACTCTTGAATGTGCTTTTCTCGCCTGAGAACTTGTTTAAAGCAGTGTACATACCCACGTTACCTTGTGGGCGGCGAGTGAAGCCACGGATGTCCTCGTAGACGTCTCGGTTGAATAACTTTAGACTCTCTCTCACCTGAGGATCGGTGTTAGAGTTAGAGCTGTAGGTTGAGTACCCCCCATACTTAGCGATTTCACGTAAGTTGGGGTCCTCGAAGTGAAGAGGGATAACATCCTCGGAAGTGGTTGACCCTGTCTTTGCGGGACGAAGGTGGAGTGCGTCAACTAAATGAGACCCCTTTCCAAGGACGTAGTGGTTGGTTGGTTCTGAAGTGAAATCTTCCATTTCGGCTCCAGAGATGAAGGAGGATAAACCCGTTGGCACGGTAAAATCGC

**PV2 RdRp:**

>MRCP6_TRINITY_DN1242_c2_g1_i1

GACGCTTTCCGTCCGCCAAGACTCTGCCGCCCTGCTCACATCTTAGATGTCAAGCATGGATACCCTTACAAATGGAATGTCAACGCTGAACCTCCGTTCTCCACTGACCCATATTTCCTTAACAACCGTAAGACATTTGGCGAGTTCATTCAGATGCACGAATATGAGCACATAGATAAGGCTGACTTTTTCCGCCGTCACCCGAACACTGGATCGCATGATTTGCTCCAGACCACCGTACCTCCAAAGTTCGGATACATGAAATCAACTGTATTCTCTTGGACCCGTAGATGGCATCACATTATCAAATCAGGATTCACTGAATCTACCGGCCTGAAAACGACAGGTTATTTTTATAACCGCTTTATCTTCCCCATGTTATTACATACCAAAACTGCAATCGTCAAGACTGATGATCCCAACAAGATGCGCACCATCTGGGGCGCCTCAAAACCGTGGATCATAGCTGAAACGATGTTCTACTGGGAATACCTTGCCTGGGTAAAACAAAACCCTGGTGTCACCCCAATGCTTTGGGGTTACGAAACCTTTACTGGCGGTTGGTTCCGACTGAACCACGCCCTGTACTGTGGCTTTATCAAACAGTCATTCCTGACCCTTGACTGGTCTCGCTTTGACAAGAAAGCTTACTTCCCACTCTTACGTAGAATTATGTACGAGGTACGCACCTTCCTCACTTTTGACGAGGGATACGTCCCCACGCATGCAGCACCCACACATCCAAAATGGGACCAAACCAAAGCAGAACGCCTTGAGCGTCTGTGGTTATGGACACTCGAAAACCTTTTCGAGGCGCCAATTATCTTACCTGACGGTAGGATGTACAAGCGCCACTTCGCAGGTATACCCTCGGGGTTGTTCACCACCCAACTCTTGGATTCATGGTATAATTATACTATGCTCGCAACCATTCTTAGCGCCTTGGGCTTCAACCCACGGACTTGCATCATTAAAGTGCAAGGTGACGATTCTATCATTAGACT

>MRCP1_TRINITY_DN1819_c0_g1_i1

TGGGAGACTACACTCTACCACCCGGACTTCTTCGCGTTAACGATGTCGCCATCAGCAACCACAAGAAAACACTGGAACACTCATTCAACAAGTATCTTTATGATCACGAAATTCGATTGATCACACAAGAATATCGACGATCCGAAATTAACATCGACTCTATATTAGCCGATTTCTTCTCCGGAGACGTTGAACCTTTTGAAATACCTTTCGACGAACACGTCGAAAACGGTCTCCAATGCATGGCCGACGCTTTCCGTCCGCCAAGACTTTGCCGCCCAGCTCACATCCTAGATGTGAAGCACGGATACCCATACAAATGGAACGTCAACGCAGAACCTCCGTTCTCAACAGACGAATATTTCCTCAAACAACGTATGACTTTTGGCAAATTCATCAGTATGCACGAATATGAACACATTGATAAACATGACTTCTTTAGACGTCATCCAAACATTGAATCACATGATTTACTCAGAACTGTCGTTCCAGCCAAATTTGGCTACCTCAAATCGATGGTCTTTTCATGGACTCGCCGTTGGCACCACATCATCAAAAGTGGTTTCAAAGACCTAACGCAACTCAATAAAACAGGATACATCTACAATCGGTTTATCTTTCCCATGCTGCTACACACCAAAACTGCTATTGTCAAATACGACGATCCTGACAAGATGCGCACCATATGGGGCGCTTCTAAACCCTGGATCATTGCTGAGACTATGCTCTACTGGGAATACATCGCTTACCTCAAACTCAACCCTGGCGTTTCGCCAATGCTTTGGGGGTATGAAACCTTCACAGGTGGATGGTTCAGACTGAACCGTACACTATATTGTGGACTTATCAAACGATCTTTCCTAACATTGGACTGGTCACGCTTCGACAAACGAGCGTACTTTCCACTGATACTTAGAATCATGCAACACGTACGTACCTTCCTCACATTTGAAGAGGGCTATGTTCCGACACATGCAGCACCGTACCACCCTCAATGGAGCCCTGAACACACACAGCGTCTCGAACGCTTATGGATTTGGACTCTCGAAAACTTATTCGAGGCACCTATCGTACTTCCCGACGGTAAGATGTTCAAACGCCACTACGCTGGAATACCTTCCGGCCTGTTCATCACTCAACTACTGGACTCCTGGTACAACTACACTATGCTCGCAACCCTCCTGAGCGCCCTTGGCTTCAACCCAAGACAGTGCATCATCAAAGTACAAGGTGACGATTCAATCATTCGTCTCAACGCCCTTATTCCTCAAGAATCACACGAGAGCTTTATGCTCCGACTTGTCGACCTGGCTACTTACTACTTCAATGCAATTGTCAACATCAAGAAGTCAGAGATAGGCAACACACTCAACGGTCGCGAAGTACTCTCCTACCGTAATCACCATGGATTACCTTGTCGTGATGAGATCACCATGCTCGCGCAATTCTATCACACCAAAGCAAGAGATCCTACACCAGAGATTACAATGGCACAAGCCGTAGGTTTCGCTTACGCAAGTTGCGGAAACCACCAACGTGTCCTCTCAGTACTACGAGACATCTATAGTTACTATCAAGCTCAAGGCGTCACACCTAACCGCGCTGGACTCACCCTCACCTTCGGAGAATCACCAGACTTAACGATGCCAGAGATACCTCTTGATCATTTTCCTACTGTAACCGAGATTCAAAATCTCCTTATTTGTAATGATTATGTAAATGAAGCACAAAACGCTCGCACTTGGCCAAGGACGTTGTTCATCAACGCCCCTGCCCAATAAAGCTGATCG

>MRCF3C_TRINITY_DN3771_c1_g1_i1

CCACTGACCCATATTTCCTTAACAATCGTAAGACATTTGGCGAGTTCATTCAGATGCACGAATATGAGCACATAGATAAGGCTGACTTTTTCCGCCGTCACCCGAACACTGGATCACATGATTTGCTCCAGACCACCGTACCTCCAAAGTTTGGATACATGAAATCAACTGTATTCTCTTGGACCCGTAGATGGCATCACATTATCAAATCAGGATTCATTGAATCTACCGGCCTGAAAACGACAGGTTATTTTTATAACCGCTTTATCTTCCCCATGTTATTACATACCAAAACTGCAATCGTCAAGACTGATGATCCCAACAAGATGCGCACCATCTGGGGCGCCTCAAAACCGTGGATCATAGCTGAAACGATGTTCTACTGGGAATACCTTGCCTGGGTAAAACAAAACCCTGGTGTCACCCCAATGCTTTGGGGTTACGAAACCTTTACTGGCGGTTGGTTC

>MRCF1C_TRINITY_DN5003_c0_g2_i1

TCAAAATACGAAAAGAAAACTTGACTGCTCTAGTTTTTAAATACCTACCCTAAATGGATTACACACATTCCGCATACCAACGAATCATTAAATGGTTCAAATCAGACCACAACTTTGAGTACGTGGGAGACTACACTCTACCACCCGGACTTCTTCGCGTTAACGATGTCGCCATCAGCAACCACAAGAAAACACTGGAACACTCATTCAACAAGTATCTTTATGATCACGAAATTCGATTGATCACACAAGAATATCGACGATCCGAAATTAACATCGACTCTATATTAGCCGATTTCTTCTCCGGAGACGTTGAACCTTTTGAAATACCTTTCGACGAACACGTCGAAAACGGTCTCCAATGCATGGCCGACGCTTTCCGTCCGCCAAGACTTTGCCGCCCAGCTCACATCCTAGATGTGAAGCACGGATACCCATACAAATGGAACGTCAACGCAGAACCTCCGTTCTCAACAGACGAATATTTCCTCAAACAACGTATGACTTTTGGCAAATTCATCAGTATGCACGAATATGAACACATTGATAAACATGACTTCTTTAGACGTCATCCAAACATTGAATCACATGATCTACTCAGAACTGTCGTTCCAGCCAAATTTGGCTACCTCAAATCGATGGTCTTTTCATGGACTCGCCGCTGGCACCACATCATCAAAAGTGGTTTCAAAGACCTAACGCAACTCAATAAAACAGGATACATCTACAATCGGTTTATCTTTCCCATGCTGCTACACACCAAAACTGCTATTGTCAAATACGACGATCCTGACAAGATGCGCACCATATGGGGCGCTTCTAAACCCTGGATCATTGCTGAGACTATGCTCTACTGGGAATACATCGCTTACCTCAAACTCAACCCTGGCGTTTCGCCAATGCTTTGGGGATATGAAACCTTCACAGGTGGATGGTTCAGACTGAACCGTACACTATATTGTGGACTTATCAAACGATCTTTCCTAACATTGGACTGGTCACGCTTCGACAAACGAGCGTACTTTCCACTGATACTTAGAATCATGCAACACGTACGTACCTTCCTCACATTTGAAGAGGGCTATGTTCCGACACATGCAGCACCGTACCACCCTCAATGGAGCCCTGAACACACACAGCGTCTCGAACGCTTATGGAGTTGGACTCTCGAAAACTTATTCG

**PV3 RdRp:**

>MRCP6_TRINITY_DN1933_c0_g1_i1

TTAAGAATTCACATTCGTCTATTATATCAAGCTTTAATCGAAGCCTCTGCACTATATCTTAACATCTCACAATGCCTCACAACGCAGTCCGAAACTACTTCGCTGAACGCATAGAACACACAGTTCGTGAAATAGCGACTTACCAGTCCCTAAATCGTGATATCGATACAACTCTTGACAATATTCAAGACAACGATTACCGTCGTCACTTTGAAGCTTTTCGTCATAACAATGACAATGAAATAAAGCGACTCGCTCTGAATAAAGAGTACTCAACATTAGTTGAAGCCTATCGAACAGATAATGCCCACAAGCACCAACCTTATGAACTACACCAGCCAATTCCCATTGGTGCAGATCCTATTCCTCGCAATCGCGAGCCCGCTCCAGGTATTAAACTCGTACCCCGCATGTACCATTATGGTCATGTTATTCGCGACCCCGAACAACCTTCTTCGTCCACTGAAACAGAACTCTCTGACGCCAGTGAAATCAATACTTCTGATGTGAAAAACTTTGGTTATCCCGTTGATCCGCAAATTCGATCCCTAATTTACAGATTATATCCAACGTATTCTCATATCATTGATGACTATTGCCGACCTATCGGAACAGCTGATGCTACTTTTAATGATTTCAATAAGGAACAGATTCATTCTGATCCAATTTCGAAAGAAAGAAAAGAACAAGTCTTAGATCTTATACATTATTTTCTAGACTCAAAACGTTATCTGCCAATCCACTTTGTCGACACTCAGCACTGTAAAACTCCTCTTGTAACTGGTACCGGTTATCATAACCGATATTCGTACAAACAGAAAGCTCATGCTAAGTATGCTCACCCTGAAGAGTATGCTCTATATCCCACCTCTAAAGGATATTTCTATAACTCCACATATGAAAGTGCTCGCACTCTCATTCACCATATTAAAATGTATGGTCTACCCAAGAATTTACATGATGCACCTCTTGACTCTTCTTCTCCCGAAGCTTATGAAAGAGACTATATTGATAAAGCTAATTCGTTTTTCAACGATTACCCGACGATGTTATTTACCCGCAACCACATTTCGAAACGATCTGGTCCTCTTAAGGTCAGACCCGTTTATGCTGTTGATGATATTTTTATCATTATCGAACTGATGCTGACATTCCCACTCACAGTTCAAGCCCGAAAATCAACGTGTTGCATAATGTATGGATTAGAAACCATTCGTGGTTCTAACCATTTTATCGACCAACTGGCTAATTCGTATTCAACGTATTTCTCACTTGACTGGTCTAGCTATGACCAACGTCTTCCAAGAGTAATAACTGACATTTATTACACCGACTTCCTCAGAAGCCTAATTGTCATTAACCATGGATACCAGCCTACGCTCGATTACCCCACTTACCCTGACCTTGATGAACACAAGATGTATTCAAGAATGGACAACCTCCTCCACTTCCTACACACATGGTACAATAATATGACTTTCATCCTACCCGATGGACTTGCATACCGCAGAACCTCTTGCGGAGTACCCTCTGGACAATACAACACCCAGTACCTCGATTCTTTTGGCAATCTATTCCTTATTATAGATGCTATGATTGAATTTGGTTTCACTGACAGTGAAATCCGAGAATTTGTTCTCCTTATTTTAGGAGATGATAACACTGGAATGACAACAATTTCTATTTATAGAATTGATAAGTTCATCATTTTCCTTGAACGCTATGCGTTAGAAAGATACAACATGGTTTTATCCAAATCAAAGTCTGTATTGACAACTCTACGCTCGAAGATTGAAACACTAGGTTATCAATGTAACTTTGGAAAACCCAAACGCTCAATTGAGAAACTAGTTGCTCAACTAATTTATCCAGAAAATGGACTTAAGCGACATACCATGGCTGCCCGCGCTATTGGAATTGCTTATGCTTCTGCTGCTCAAGACTACATATTTCATTCATTTTGTCATGATATATACAATTTGTTTCAGTCAGATTATAAACCAGATGCTCGCGCCAATCTGTTCTTCCAACGACAAGTAATGCACAATCTTGAAGAAGGAATTCCAGACCTCGCAACATCTACTGTGCCGATATTTCCATCATTTTGTGAAGTTGAAAGTGTTTACGCTGCCTACAAAGGCCCGCTCAACTATAGACCCAAGTGGTCTACACCTCATTTCCTCCATGATCCAGATATCACGCCCCCCAACTCCAAAACTATGCTCGAGTATGAACGAGAGAATGGCATCCCTCCGAAATCCGCGCCTACTTTTGAAACGGTAGTGCCTAGCACATGAAATTTTCCGTGTATTCTTTTTG

>MRCP1_TRINITY_DN5413_c0_g1_i1

GGCCCCCCCTCGAGATATTAAGAATTCACATTCGTCTACTATAACAAGCTTATATCAAAGCCTCTGCACTATATTTACTTAACGTTCACAATGCCTCACAATACAACTCGTGACCTTCATGAGGAACGTATTCATCATACAAGAAAGCTTCTAGCTTTAGCTCAGAAGTACAATTATGATCCAGAAGCAATCCTTGAACACACTCAAGATACTGATTATCGTCGATATTTTAATGCTATGCGTTATAATCCTAACAACGATACAAGACTTCTTGCGCTTAACAAGGAATATTCAACTTTAGTTGAAGCTTACCGAACTGACAATGCTCACAAGCATCAACCATATGAGCTACATCAACCTATTCCTATAGGTGCTGCACCCATCCCTGAATCAAGAATGCCTGCCCCAGGTATTAAGTTAGTTCCTTTAATGTATCATTACGGACATGTCATACATGATCCAGTTGAAAACGATCCCCATAACACTGATCCCGAGAATGATTTCTTTGGCATCAATGCTGATGCACCTACTAGATTTGGCTACCCTTTGGATAAACGTATTTACAACAAAATCGTCCAAATGTATCCTGAATATCTAAAGGTGATTCATAGTTATTGTCGACCTATTGGCTCAGTCGAAGCTACTTTTGCTGATTTCAATAAAGAGCAAGTACCATCTGCTCCTATAAATGAAGAGAGGAAACAAGCTGTTTTAAAGCACGTTTTCAAGTTCCTTGACGCAAAACCTTATTTGCCGATTCATTTTGTTGACACTCAATATTGCAAGACACCTCTTGTTACTGGAACTGGATATCATAATCGATATAGTTTCAAACAGAAGGCACATGCGAAATACTCTCATCCAGAGCTTTATGCAAATAGCCCCACTTCAAAAGGCTACTTTTATAATGCAACCTATGAGAATGCTCGTACGCTAGTTCATCATATTAAAAAGTATGGACTACCATTTAACATGCAATACTCTCATTCCGAAAACCTTTTGGAAGAAGAGAATATCAAAGACTACATCAACAAGGCTAATTCATTCTTTAATGATTATCCAACGTTATTATTTACCCGCAACCACATTTCGAAGCGAGATGGTCCTCTCAAAGTACGTCCAGTCTACGCAGTTGATGATTTATTCATCATAATTGAACTGATGCTGACTTTTCCGTTAGTTGTTCAAGCTCGAAAATCTACCTGTTGCATAATGTATGGACTAGAAACCATTCGTGGTTCTAACCATTACATATATCGTAAAGCAAGCAAGTATTCAACATTCTTCTCACTTGATTGGTCAAGCTATGATCAACGATTACCAAGAGTGATTACCGACATATTCTACACTGATTTTCTCAGAAGTTTAATCGTCATAAACCAAGGATACCAAGCTACTTACGAATACCCAGACTATCCTGATCTTAATGAACATAACATGTACTCAAGAATGGACAATTTACTCCATTTTCTCCACACATGGTACAATAACATGACCTTCCTACTCCCTGATGGTTATGCTTACCGCAGACAATATTGTGGTGTACCCTCTGGACAGTACAATACCCAGTACCTAGATTCATTTGGAAATTTATTCTTAATAATAGATTCCATGATTGAATTTGGTTTCTCTGAAACTGAGATTGACGAATTCGTCCTTCTCGTTCTTGGTGATGACAATACTGGCATGACGACTATACCAATTAGTCGAATTGACAAGTTCATTAATTTTCTTGAAGAGTATGCGCTAAGACGATACAACATGGTCCTTTCATCAACGAAGTCCATTTTGACGACCCTGCGCTCCAAGATTGAAACACTCGGTTATCAATGTAATAATGGTTCTCCTAGAAGACCGATTGATAAACTAGTTGCTCAACTCTGCTATCCAGAAAATGGTATTAAGCCTCATACTATGGCCGTTCGTGCTATAGGCATTGCTTATGCCTCTGCTGCTCAAGATGTTACATTTCATTCCTTTTGTCATGATGTGTACAACCTATTCCGATCAGAATATAAACCAGACGTTCGCGCGAATCTCTACTTTCAACGCCAGATTATGCATAATCTAGAAGATGGAATTCCAGATCTCGCCACGCCCACAGTGCCGATGTTTCCATCGTTCCGTGAAGTACAGCATATGTACTCAAAGTACCAAGGCCCACTATCCTACTATCCGAAGTGGAACCGAGCTTATTTCATGAATGATCCTGATATCACGCCTAACCCCCCCAAGACTATGAAGCAATATGAAGAAGAGCATGATCTTACTCCATTAGCCGCTCATACTTTTGAAACGGTAGTGCCTAGCACTTAGAAAATTTTCCGTGTATTCTTTTTGATTTTTATCAAATTGTAACTCCGGTTATAACCAAAAATTATAAAAAAAAAAAAAAAAAAAAAAAAAAAAAA

>MRCF3C_TRINITY_DN12756_c0_g1_i1

CAAATTTCTACACACTAAGAAGTACCTACCCCTACACTTTGTTGACACAGGATTCGCTAAGACTCCGTTAAACACAGGAACAGGATACCACAATAGGCATTCCTTCAAAATGAACGCTCATGCTAAATACTCACACCCCGACGAATATGCTGATAAACCTACAAGTAAAGGTTTTTACATAAATGCATTTCTAGAACAAGCTCGAACCATCGTGCACTACATCAAACACTTCGGCTATCCATACTCAACTCCAAATGGAACAATAACGCCAGAAATGATGAAGACGAATCTAGACAAATTCTTCAATGAGTATCCAACTATATTATTCACCAGAAACCACATCTCAAAAATTGAAGGCCCACTAAAACAAAGGCCCGTATATGCTTGCGACGACTTGTTTATAACAATTGAATCTATGTTGACTTTCCCGCTCCTAGTACAAGCACGCAATCCACAAAATTGCATCATGTATGGACTAGAAACAATCCGCGGAGCAAACCAATACCTAGATCTTATTGCTCAGAGCTATAAGTCATACTTTACCATCGATTGGTCAAGTTTTGATCAAACTCTCCCTAGAGAGATTACAAGCTCATACTACAACGTGTTTCTCCCACAATTGATAATATATAACAAAGGTTACCTCCCAACTTATGAATACCCTACACATCAAGGAATTGATGATGAAAGGATGTATAGTATGTTAAGTAACCTCTTAAACTTCCTACACTGCTGGTATGTCAATATGACATTCCTAAGCCAAAATGGTTATGCATACAGAAGAGTCCACGCTGGACTTCCTTCAGGTATGTTAAATACCCAGTACCTTGACAGTTATTGCAACCTCTATGTTATTATAGATGCATTAATTGAATTCGG

>SSDW2B_TRINITY_DN11537_c0_g1_i1

AAGCACGTTACTATGCTATTTACTCGCAACCACATCTCCGACAGAGATGGAAACCTCAAACAACGTCCTGTTTACGCAGCTGATGACTTCTTCATCATGTGTGAATTAATGACTACGTTTCCACTCCATGTCATGGCCCGATACCCTATCAATGGTATCAAGTCTGCAATCATGTATAGCTTCGAAACTATCAGAGGATCCAACCACTACTTGGACTCACTCGCTCAATCATTTCGATCCTTTTTTACAATAGATTGGTCTTCATTTGATCAACGCGTTCCACGCTTAATCACTGACCTTTTCTTTACTGATTATCTCCGCAGTCTACTCATCATAAACCACGGCTACCAACCTACTTACGAGTATCCCACCTACCCTGATCTAACTGAACACGATCTCTACCACCGTATGTCAAATTTACTTAATTTCATGCACATCTGGTATAACAATATGGTTTTCGTCACCGCTGACGGATTCGCATACCTACGATCTACCGCAGGCGTACCTTCTGGTATTTTAAATACCCAGTACATTGATTCATTTGCTAACGCATTCCTCCTCGTTGATGGACTTCTTGAATATG

**PV4 RdRp:**

>MRDW2_TRINITY_DN2343_c0_g1_i1

TAGCCTTCCACGCGTGAGGGCATTCGACGTCAACACACAACTTGACTTAATACCATACGAATCCAGCTCATCGGCAGGTTACGGCTATATTGGAGCGAAAGGCCCATTCAGAGGCCCAAACCACCTCAGAGCCATGGCCAGAGCAAAAGCTACCATTTACGGAGCTTGCAGTTCAGATGGACCAGGTATTGAGCATCAGTTACGCACTGTTGTTCCTGACGTTGGATATACTCGCACTCAGTTAGCAGATATCGCAGAGAAGACAAAAGTACGCGGCGTGTGGGGAAGAGCATTCCACTACATTTTACTTGAAGGTACTTCGGCCAGACCGATCCTTGAAGCATTTCAGCAAGCGGACTCCTTCTATCAGATAGGAAGCGATCCAACTGTTAACGTCCCAGAGAAACTAGGGCAATTAAGTCGCATGTGCAATTGGCTATACGGCATAGATTGGTCCAAGTTTGATGCAACAGTGAGCAGATTTGAAATCAATACAGCTTTCGACATTTTAAAGACAATGGTTATATTTCCAAACTTTATTACAGAACAAGCGTTTGAATTCTCAAGACAGATCTTCATACACAAGAAGATATGCGCTCCTGACGGCAAAATATACTGGGCTCATAAAGGTATCCCATCGGGCAGCTACTATACATCAATAGTAGGATCCGTCATCAACAGAATTCGTGTAGAATACATATGGAGACTTCAATTTAACAGAGGTCCTGATAAATGTTACACGCAAGGAGATGACTCACTCGTCGGCGATAAAGATTACTACAATCCGGAATTGATGGCTACTAACGCAGCCCCATTAGGATGGAGAATCAACGCTGACAAGACTGTCTGTTCAAGATTACCAAGTGAAGTAACTTTCCTAGGCAGGACTACCACTGGTGGACTTAACCAAAGAGATCTTATTAGATGTCTAAGGTTGCTCATCTTACCAGAGTATCCAGTTGAATCGGGAAGGATTTCAGCATATAGAGCA

>MRCP6_TRINITY_DN1087_c0_g1_i1

AATCGCGGAACCTAAACTTAACAGTTGAGGTCGCAAGATTTCGGTCGTGAAAATCATTTCATTACAGCCAGTCTTCATCATGAGAAGATTGCGCAACCCTTTAGCTGGTTACATCTACACAGGATTCAACGCGGATCTTGAAGAGACAAATCAGCACCACACAAACGTTATTCGACGGGAAACCGCCGTAACGTACAGAGATGAGTTTGCATTGCAAGCCATCCAAGATCTGGATTACAGACAGTATGAGCAACATTTACAAGGTTGGTCACGTAGTTACTATACTCCTGAGAAGCATCAGGAAGCAATTATGCAATATGCCTATCCAGACATACCAGTTCAAGCCATTAAAATGGACGTATACAACGATTGTATCGCCCAGGTTCAGAACGAATTTCGTAGCCTTCCAAACGTGAGGGCCTATAGCGTATTAACCCAATTAGATTTGGTAAAATACAAGTCATCATCGGCGGCCGGTTACGGATACCAAGGTACGAAAGGAATCCCAGGTGAGTTAAATCACACTAGGGCTATTTCCCGAGCCAAGGCGATATTATGGTCAGCCATCGCAGCAGACGGTGAAGGCATCGAACATGTCATCAAGACTTCAACGCCAGACATAGGCTATACACGTACCCAGCTTACAGATCTCACTGAGAAGACGAAAGTCAGACAAGTATGGGGTAGAGCTTTTCATTACATCCTCCTAGAAGGATTGGTCGCAGACCCATTCATTCAGGCGGTTATGGAGAACGATACATTCATCCATGCTGGACGAGATCCAACATTAAGCGTGCCACAATTGCTATCGCAAGTAGCGGAAACATGCGACTGGATATATTCATTAGATTGGAAACAGTTTGATGCAACAGTTAGTAGATTCGAAATCGAGTCAGCTTTCTCAATTATCAAGTCCAAGACGATCTTCCCTGATAGAGAAACGGAAGATGCTTTTGAGATAACTAAACAGTTATTCATTCATAAGAAAGTCGCCGCACCAGACGGTAAGATATATTTTGCGCACAAAGGAATTCCATCAGGAAGTTATTACACTTCACTAGTCGGATCTATTGTTAATAGACTCAGAATTGAGTATCTATTCAGACTAATCCTAGGACGAGGTCCAAAGATTTGTCATACATTAGGAGACGATTCACTAGTAGGTGATGACGAGTTGACGGTCCCAACAACATTTGGCGTTATCGCTAATCACATTGGTTGGTACTTCAACACTGAAAAGACGGAGTACTCGAGAATACCGGAGATGGTCACATTTCTAGGTCGATCCTACAAAGGTGGACTCAACGTACGTGATCTTAAACGATGTTTGAGGTTACTAGTCTTTCCTGAATATCCTGTTACGACAGGACGAATTTCAGCATATAGAGCTAAATCGATTGCAGAAGATTGCGGACACATCAGTGATCTACTAAATCGAGTAGCAGCTAGATTAAAGCGTCAGTACGGACTCGCTTCTGAAGAAGAAGTTCCCATCTATTTTAAGCGTTATTTACCTTTTATGTAATTTTACATTTAAGTTATTTATGTTTTAATAATAAGACGTGGAGGTTACA

>MRCF1C_TRINITY_DN16662_c0_g1_i1

GAGAAGAAGCTGTCACTTACCGTGACGAATTCGCTCTCAGCGTATTACATGATTTATATCCGCGCATATATGAGCAATACTTAGAAGGATGGTCAAGAAGCTATTATTCAAAAGAAATGCACATGAAAGCAATTATGCAATATGGATTACCAGACATCCCACCAACCCAGGTGGATCAAGATTGTTACAAACAATCAATCAACACTATTTTAGAATTGTTGAAAGGCCTACGCAATGTACGAGCTTACGACGTTTTAACTGAATTAGATAAAGTAGATTACGAATCTACCTCATCAGCAGGTTACGACTATATAGGTGTCAAAGGACCCATAGATGGTGAAAATCATAAAAGGGCAATACGACGAGCAAAAGCAACCTTATGGTCAGCAATCAATTTATCTGATCCAGGTATGGACCATGTCATCGAGACATCAGTCCCAGACGTCGGTTACACCCGAACACAGTTAACGGATCTTTATGAAAAGACTAAAGTCAGAGGAGTATGGGGTCGAGCATTCCATTACATCTTACTCGAAGGAACAGTGGCTAAGCCATTGTTAGACTATTTCACCCGTGGAGAGACATTCTTTCACATAGGGCAAGATCCAACAATCAGCGTACCA
